# Unveiling hydrocerussite as an electrochemically stable active phase for efficient carbon dioxide electroreduction to formate

**DOI:** 10.1038/s41467-020-17120-9

**Published:** 2020-07-08

**Authors:** Yanmei Shi, Yan Ji, Jun Long, Yu Liang, Yang Liu, Yifu Yu, Jianping Xiao, Bin Zhang

**Affiliations:** 1 Institute of Molecular Plus, Department of Chemistry, School of Science, Tianjin University, Tianjin, 300072 China; 2 Tianjin Key Laboratory of Molecular Optoelectronic Science, Collaborative Innovation Center of Chemical Science and Engineering, Tianjin, 300072 China; 3 State Key Laboratory of Catalysis, Dalian Institute of Chemical Physics, Chinese Academy of Sciences, University of Chinese Academy of Sciences, Dalian, 116023 China; 4 School of Science, Westlake University, Hangzhou, 310024 China; 5 Analysis and Testing Center, Tianjin University, Tianjin, 300072 China

**Keywords:** Electrocatalysis, Sustainability, Electrocatalysis

## Abstract

For most metal-containing CO_2_ reduction reaction (CO_2_RR) electrocatalysts, the unavoidable self-reduction to zero-valence metal will promote hydrogen evolution, hence lowering the CO_2_RR selectivity. Thus it is challenging to design a stable phase with resistance to electrochemical self-reduction as well as high CO_2_RR activity. Herein, we report a scenario to develop hydrocerussite as a stable and active electrocatalyst via in situ conversion of a complex precursor, tannin-lead(II) (TA-Pb) complex. A comprehensive characterization reveals the in situ transformation of TA-Pb to cerussite (PbCO_3_), and sequentially to hydrocerussite (Pb_3_(CO_3_)_2_(OH)_2_), which finally serves as a stable and active phase under CO_2_RR condition. Both experiments and theoretical calculations confirm the high activity and selectivity over hydrocerussite. This work not only offers a new approach of enhancing the selectivity in CO_2_RR by suppressing the self-reduction of electrode materials, but also provides a strategy for studying the reaction mechanism and active phases of electrocatalysts.

## Abstract (web summary)

While electrochemical CO2 reduction represents a renewable means to produce high-value products, catalyst transformation may compete with desirable processes. Here, authors prevent catalyst self-reduction during CO2 electroreduction and show stable, formate-selective performances of hydrocerussite.

## Introduction

Electrocatalysis has emerged as a promising technology for renewable energy conversion and storage^1–4^, where a key factor is the development of electrocatalysts with higher activity, better selectivity, longer stability, and lower cost^5,6^. While the activity and stability are the two ends of a seesaw, the high activity of electrocatalysts often cannot be sustained for a long time, mainly because of the undesirable reconstruction of electrode surfaces. With the development of in situ characterization techniques, researchers have observed the structural transformation of electrocatalysts during the electrolysis^7^. Many efforts have been devoted to exploring the real active species derived from the pre-catalysts. For instance, metal oxyhydroxides, derived from the oxidative transformation of metal chalcogenides and pnictides, are found to be the active species for the oxygen evolution reaction (OER), promoting the rapid developments of highly efficient electrocatalytic materials for OER^8–10^. Thus the exploration of the real active species is of great importance for the rational design and synthesis of advanced electrocatalysts with high intrinsic activity.

Electrochemical CO_2_ reduction reaction (CO_2_RR) can convert the greenhouse gas (CO_2_) to various value-added chemicals^11–16^, while the competing hydrogen evolution reaction (HER) is usually more favorable on many metallic electrodes. Although many high-valence metal electrocatalysts are proved to be reduced to zero-valence metal at CO_2_RR condition, such as BiOI^17^, PbO_2_^18^, SnO_2_^19^, CuO^20^, In_2_O_3_^21^, it was found the residual metastable metal oxides are tightly correlated with the high selectivity towards CO_2_RR^22–24^. However, with the self-reduction occurring, HER becomes more and more dominant, leading to a diminished CO_2_RR efficiency. Besides, many complex electrocatalysts also undergo unavoidable dissociation and self-reduction under cathodic working conditions. For example, a Ni(II) benzenedithiol complex was revealed to be completely transformed to Ni nanosheets under cathodic conditions^25^. A Cu(II) phthalocyanine complex undergoes reversible conversion to metallic Cu clusters, which acts as the active species in CO_2_RR^26^. The self-reduction of electrocatalysts and the desirable CO_2_RR are actually competitive processes. To achieve high selectivity, developing electrocatalysts with predominant CO_2_RR over self-reduction is highly desirable.

Herein, we report a scenario to make effective use of the electrochemical structure evolution of a complex pre-catalyst, tannin–lead(II) (denoted as TA–Pb), toward high formate selectivity in CO_2_RR. The TA–Pb pre-catalyst performs a formate Faradaic efficiency (FE) of up to 96.4 ± 0.9%. Through a set of comprehensive characterizations, it is identified that TA–Pb is transformed to cerussite (PbCO_3_, denoted as PCO) at first, then sequentially to hydrocerussite (Pb_3_(CO_3_)_2_(OH)_2_, denoted as PCOH) at steady state, which essentially serves as the active phase for highly selective formate production. In addition, we also synthesize pure cerussite nanoparticles and hydrocerussite nanoplates to confirm the transformation mechanism and the key role of hydrocerussite in CO_2_RR, respectively. Moreover, the density functional theory (DFT) calculations also reveal the high formate selectivity, which can be attributed to the appropriate binding strength with HCOO* on hydrocerussite. All the results unveil hydrocerussite as a highly selective and stable phase for formate production in CO_2_RR.

## Results

### Electrochemical performances and transformation of TA-Pb

**Fig. 1:**
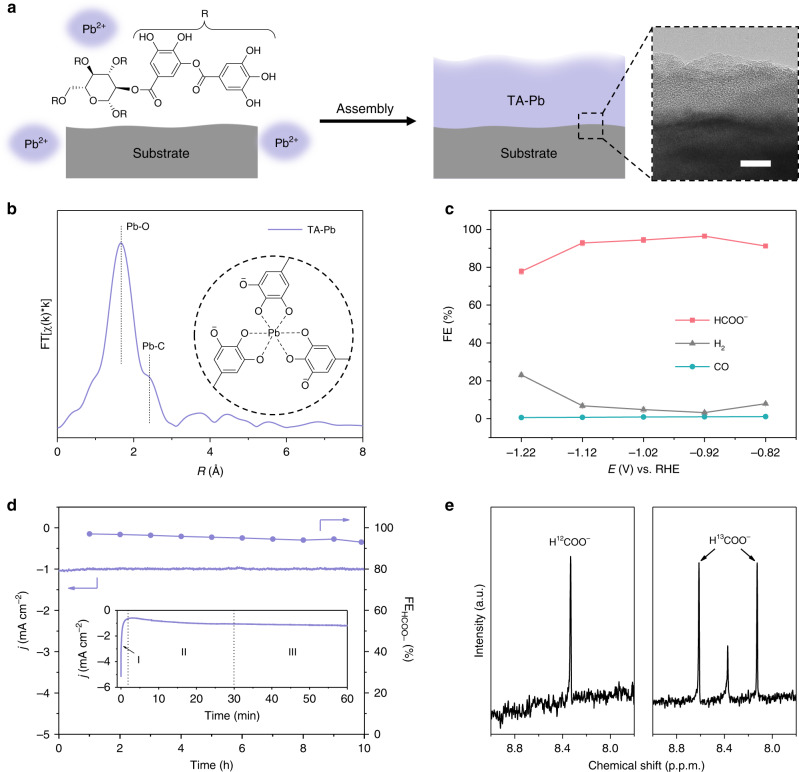
Preparation, characterizations and electrochemical performances of TA–Pb. **a** A scheme illustrated for the TA–Pb preparation. The inset is the HRTEM image of single-layer TA–Pb. Scale bar, 10 nm. **b** Pb *L*_3_-edge EXAFS spectrum of TA–Pb. The inset is the proposed structure of TA–Pb. **c** FEs of 3-layer TA–Pb at steady state under different potentials. **d** *j-t* curve and corresponding formate FE of 3-layer TA–Pb at steady state (stage III in the inset) under −0.92 V. The inset is the *j-t* curve of the as-prepared TA–Pb, which can be divided into three stages labeled as I, II, and III. **e** ^1^H-NMR spectra of formate by using ^12^CO_2_ and ^13^CO_2_ as feedstocks by using 0.5 M Na_2_SO_4_ as the electrolyte to eliminate the interference of NaHCO_3_. Error bars correspond to the standard error of the mean.

The tannin–lead(II) (TA–Pb) complex film is prepared by simply mixing Pb(II) and tannin aqueous solution in the presence of carbon fiber paper (CP) as the substrate (Fig. 1a). By examining with a high-resolution transmission electron microscope (HRTEM), the coordination of TA and Pb(II) forms a layer of amorphous film contacting closely to the substrate with the thickness of ~20 nm for single-layer TA–Pb with CP as the substrate (inset in Fig. 1a, Supplementary Fig. 1 and Supplementary Note 1). The extended X-ray absorption fine structure (EXAFS) exhibits clear coordination of Pb–O and Pb–C for a five-membered ring in a chelate ligand (Fig. 1b)^27^, suggesting the 6-coordinated Pb(II) ions as reported before^28^.

The electrochemical activity was tested in a standard three-electrode system with 0.5 M NaHCO_3_ as the electrolyte (Supplementary Fig. 2). Before CO_2_RR tests, all the samples were pre-treated under −0.92 V for 30 min to reach the steady state. Liquid products were detected by nuclear magnetic resonance (NMR), while the gaseous ones were detected by gas chromatography (GC). The products in CO_2_RR with single-layer TA–Pb as pre-catalyst are formate, CO, and H_2_, which is in accordance with other reported Pb-based electrocatalysts^29–31^. And −0.92 V vs. reversible hydrogen electrode (RHE) is the optimum potential to achieve the highest formate FE (Supplementary Fig. 3 and Supplementary Note 2). The formate FE can be further improved by increasing the layers of the TA–Pb, and the 3-layer TA–Pb complex shows the highest formate FE of 96.4 ± 0.9% at the optimum potential of −0.92 V with a small amount of H_2_ and negligible CO. This is almost the highest value among Pb-based electrocatalysts (Supplementary Table 1). The formate FE remains above 90% at a wide potential range from −0.82 to −1.12 V (Fig. 1c). The result of inductively coupled plasma-mass spectrometry (ICP-MS) exhibits an extremely low Pb loading of ~25 μg cm^−2^ for 3-layer TA–Pb, thus leading to a high formate turnover frequency (TOF) of 0.055 s^−1^ at the optimum potential of −0.92 V. Compared with the TA–Pb pre-catalyst, Pb foil displays much lower formate FEs of <40% (Supplementary Fig. 4). It is also noticed that the *j-t* curves of the as-prepared 3-layer TA-Pb can be divided into three stages, i.e., stage I (0–3 min) with current density sharply declining, stage II (3–30 min) with current density increasing mildly, and stage III (>30 min) with almost constant current density (inset in Fig. 1d). These stages indicate the structural evolution of TA–Pb, whose details will be discussed later. At a steady state, the derived active species performs excellent stability of both formate FE and current density for 10 h (Fig. 1d). Labeled ^13^CO_2_ was used to verify the carbon source of produced formate in control experiments under the optimum potential. As shown in the ^1^H-NMR spectrum in Fig. 1e, the H^12^COO^−^ shows the chemical shift only at 8.35 ppm. The appearance of two new peaks at 8.61 and 8.13 ppm indicates the formation of H^13^COO^−^, reflecting that carbon in formate is from CO_2_ rather than the decomposition of the complex.

**Fig. 2:**
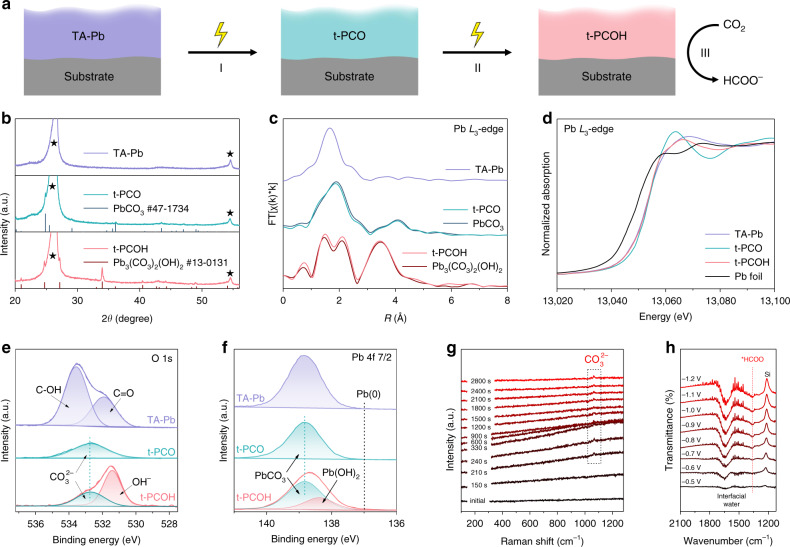
Transformation of TA-Pb under cathodic potential. **a** Illustration of the transformation from TA-Pb to t-PCO and t-PCOH. **b** XRD patterns, **c** Quasi-in-situ Pb *L*_3_-edge EXAFS, **d** XANES spectra, **e** O 1s, and **f** Pb 4f 7/2 XPS spectra of TA–Pb, t-PCO and t-PCOH. The XRD peaks from the substrate are labelled by black stars. **g** Time-dependent in situ Raman spectra of TA–Pb under CO_2_ bubbling. **h** Potential-dependent in situ ATR-FTIR spectra of hydrocerussite with CO_2_ bubbling. The bands around 1640 and 1220 cm^−1^ are attributed to the adsorbed interfacial water and the vibration of Si–O from silicon as the reflection window, respectively^51^.

Based on the three stages in Fig. 1d inset, we have captured another two different states derived from TA–Pb in the electrolysis (Fig. 2a). The X-ray diffraction (XRD) patterns show that the two intermediate phases are cerussite (PbCO_3_) and hydrocerussite (Pb_3_(CO_3_)_2_(OH)_2_), respectively (Fig. 2b). And the phase of hydrocerussite maintains after a long-term test (Supplementary Fig. 5). The TA–Pb derived PbCO_3_ and Pb_3_(OH)_2_(CO_3_)_2_ are denoted as t-PCO and t-PCOH hereafter. Their corresponding HRTEM images show the decreasing thickness and increasing crystallinity along with the cathodic electrolysis (Supplementary Fig. 6). The structures of these intermediates were further identified by quasi-in-situ X-ray fine structure (XAFS). The EXAFS spectra of t-PCO and t-PCOH match well with the references cerussite and hydrocerussite, respectively (Fig. 2c). The similar Pb *L*_3_-edge in the X-ray absorption near edge structures (XANES) of different stages indicates that the valence state of Pb remains unchanged during the electrochemical CO_2_RR measurements (Fig. 2d).

The X-ray photoelectron spectra (XPS) of TA-Pb clearly show the organic functional groups of tannin molecules in the C 1s and O 1s spectra (Fig. 2e and Supplementary Fig. 7). However, these peaks of organic functional groups completely disappear in the t-PCO, suggesting the decomposition of the TA-Pb complex in stage I. The symmetric O 1s peak of t-PCO at 532.7 eV suggests the O element only exists in the form of CO_3_^2−^ ^32^. However, in t-PCOH, the appearance of a new peak represented OH^−^ confirms the formation of hydrocerussite^32^. Similarly, the symmetric peak at 138.8 eV is attributed to PbCO_3_ in the Pb 4f 7/2 spectra of t-PCO (Fig. 2f), while the peak at 138.3 eV in t-PCOH represents the formation of Pb-OH^33^. It should be pointed out that no Pb(0) is found throughout the whole process. And the cyclic voltammetry (CV) curve of t-PCOH shows no redox peak in the tested range, firmly verifying the stability of hydrocerussite in CO_2_RR (Supplementary Fig. 8).

The transformation of the TA-Pb complex was further investigated by Raman spectroscopy and Fourier transform infrared (FTIR) spectroscopy. The time-dependent in situ Raman spectra are shown in Fig. 2g. It is clearly shown that the fluorescent intensity from the ligand tannin ascends at first and then descends with the time prolonging (Supplementary Fig. 9,10), indicating the TA-Pb complex undergoes a part-to-whole dissociation^34^. A small peak at 1065 cm^−1^ appears after electrolysis for 210s, which is attributed to C–O symmetric stretching vibration of CO_3_^2−^ ^35^, indicating the formation of t-PCO. Because of the similar Raman spectroscopy shape of cerussite and hydrocerussite in this region^36^, the formation of t-PCOH cannot be distinguished from Fig. 2g. It should be also noted that the peak of CO_3_^2−^ appears as early as the ascending process of fluorescence, which means that the dissociation of TA-Pb and the formation of t-PCO are accompanying rather than separate and sequential. Moreover, in ex situ FTIR spectra, the disappearance of the complex peaks again confirms the dissociation of the complex (Supplementary Fig. 11). In situ attenuated total reflectance-Fourier transform infrared (ATR-FTIR) spectroscopy was employed to investigate the reaction path of hydrocerussite. As shown in Fig. 2h, with the applied potential decreasing, the intensity of bands at ~1360 cm^−1^ gradually increases (Supplementary Fig. 12 and Supplementary Note 3), which can be associated with the vibration of O–C–O in the two-oxygen bridge-bonded formate species (*HCOO)^37^, confirming the *HCOO pathway of hydrocerussite (Supplementary Fig. 13). No obvious band associating with CO* can be found in the region of 1900–2100 cm^−1^, indicating that the formation of CO on hydrocerussite is almost inhibited^38^, which is consistent with the above experimental FE results. All the above results confirm that the TA–Pb complex electrochemically dissociates and transforms into cerussite and hydrocerussite sequentially, in which the valence state of Pb remains +2 during the whole electrolysis, suggesting hydrocerussite to be the active species for electrochemical CO_2_RR via the pathway of *HCOO as the intermediate.

### Performances of as-prepared cerussite and hydrocerussite

**Fig. 3:**
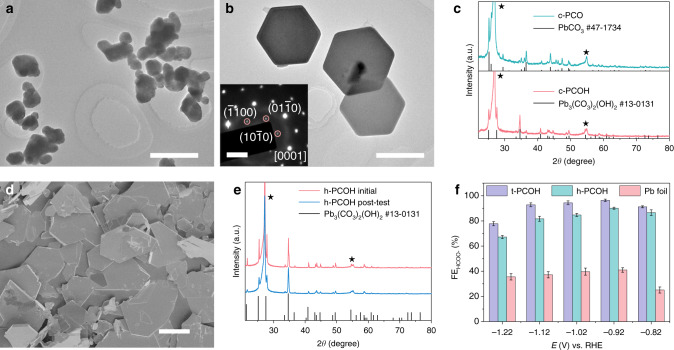
Performances of c-PCO and h-PCOH under CO_2_RR condition. **a** TEM image of c-PCO. Scale bar, 500 nm. **b** TEM image of c-PCOH transformed from c-PCO. Scale bar, 500 nm. The inset is the corresponding SAED pattern. Scale bar, 2 1/nm. **c** XRD patterns of c-PCO and c-PCOH. The peaks from the substrate are labelled by black stars. **d** SEM image of the as-prepared h-PCOH. Scale bar, 10 μm. **e** XRD patterns of h-PCOH before and after the CO_2_RR test for 5 h. The peaks from the substrate are labelled by black stars. **f** Comparison of formate FEs of t-PCOH, h-PCOH, and Pb foil at different potentials. Error bars correspond to the standard error of the mean.

To further investigate the transformation of cerussite to hydrocerussite, we prepared cerussite nanoparticles (denoted as c-PCO) and studied their behavior under the same conditions of CO_2_RR. The TEM image of the as-prepared c-PCO shows the irregular nanoparticles (Fig. 3a and Supplementary Fig. 14a). After tested at −0.92 V in the presence of CO_2_ for 30 min, it is found that the c-PCO nanoparticles completely transform to monocrystalline hexagonal nanosheets (Fig. 3b and Supplementary Fig. 14b). The corresponding selected area electron diffraction (SAED) pattern shows the dominant exposed facet of (0001) (inset in Fig. 3b). XRD pattern of the hexagonal nanosheets matches well with hexagonal hydrocerussite without any impurity (Fig. 3c), and the nanosheets is named as c-PCOH. XPS and ex situ Raman spectra also confirm the phase change from cerussite to hydrocerussite (Supplementary Figs. 15, 16). By studying the transformation process, it is found that the c-PCO nanoparticles first convert to cerussite nanoprisms via an Ostwald ripening process^39,40^, then to hexagonal hydrocerussite nanosheets through the selective etching of CO_2_ and the insertion of OH^−^ (see more details in Supplementary Figs. 17–20 and Supplementary Notes 4–7).

The hydrocerussite nanoplates (shorted as h-PCOH) were also fabricated to estimate their CO_2_RR performance. The SEM image of the as-prepared hydrocerussite nanoplates performs *quasi*-hexagonal nanoplates with the diameter of several microns (Fig. 3d). After the CO_2_RR measurements, all the results of SEM images (Supplementary Fig. 21), XRD patterns (Fig. 3e), XPS spectra (Supplementary Fig. 22) and ex situ Raman spectra (Supplementary Fig. 23) indicate that the morphology, structure, and composition of the h-PCOH remain unchanged, confirming that hydrocerussite is stable and the active species for CO_2_RR. When compared the CO_2_RR performances of h-PCOH with t-PCOH, it is found that h-PCOH generates the similar products of dominant formate, a small amount of H_2,_ and trace CO (Supplementary Fig. 24a). Specifically, h-PCOH performs the formate FE of 90.1 ± 0.8% at −0.92 V with the stability of over 10 h (Fig. 3f and S24b). The slightly lower FE of h-PCOH may originate from the poor coverage of the relatively large h-PCOH nanoplates (Supplementary Fig. 21), leaving part of the carbonaceous substrate exposed to give off much H_2_. The result, in turn, confirms the high coverage of in situ formed t-PCOH. Moreover, limited by the high mass loading of h-PCOH (4.01 mg cm^−2^ for Pb), its TOF is calculated to be only 6.8 × 10^−4^ s^−1^, which is 80 times lower than that of t-PCOH, further indicating a high catalytic efficiency of the complex-derived hydrocerussite.

### DFT calculations

To understand the chemical origin of high formate selectivity in CO_2_RR over hydrocerussite, DFT calculations were performed. The hydrocerussite models are structured, and the optimized lattice parameters of 5.28 × 5.30 × 23.68 Å are in good agreement with the experiments (Supplementary Fig. 25a)^41^. As observed above, hydrocerussite prefers to expose the (0001) surface. In this way, there are three possible terminations of hydrocerussite (0001), denoted with Layer A, B, and C, as shown in Supplementary Fig. 25b. The B-termination is thermodynamically most stable with the surface energies (*E*_s_) of −0.24 eV nm^−2^ relative to A (*E*_s_ = −0.04 eV nm^−2^) and C (*E*_s_ = 2.64 eV nm^−2^). Besides, since hydrocerussite is in situ produced from the complex pre-catalyst, two kinds of vacancies (CO_3_^2−^ or OH^−^ removal) are considered herein. As a consequence, six possible active sites on hydrocerussite are examined to compared with the reference metallic Pb(111), including perfect A, B, and C terminations, denoted as A-p, B-p, and C-p, and defected A, B, and C ones, denoted as A-d, B-d, and C-d (Supplementary Fig. 25b). Then we calculated the adsorption energies of all the possible intermediates in CO_2_RR to producing H_2_, CO, and formate, respectively. According to the result of in situ ATR-FTIR spectra, *HCOO adsorption energy is chosen as the descriptor to establish the correlation between different active sites and adsorbates, as shown in Supplementary Fig. 26.

**Fig. 4:**
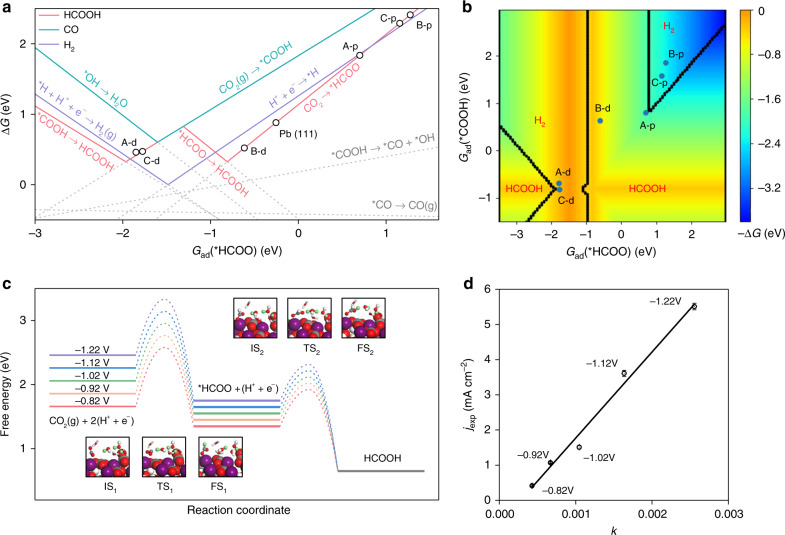
Theoretically simulated the activity and selectivity of hydrocerussite. **a** Reaction free energies of involved elementary reactions plot versus *G*_ad_(*HCOO). **b** Two-dimensional map of activity and selectivity for CO_2_RR. Different regions represent different products, and colors represent the negative Δ*G* of GDS measured as the label on the right side. **c** Free energy diagrams of CO_2_RR into HCOOH under different experimental potentials; insets are structures of initial (IS), transition (TS) and final (FS) state of CO_2_ protonation (1) and HCOOH formation (2); purple, red, gray, and white atoms are Pb, O, C, and H, respectively; green atoms represent H that participate in the reaction. **d** Experimental partial current density of formate (*j*_exp_) versus theoretical charge transfer rate *k* (10^−17^ C ∙ s^−1^ ∙ site^−1^). Error bars correspond to the standard error of the mean.

The reaction free energies (Δ*G*) of all possible elementary steps are calculated and plotted versus the adsorption free energy of *HCOO [*G*_ad_(HCOO)] (Fig. 4a), where the solid lines represent the Δ*G*-determining steps of HCOOH, CO, and H_2_ production. The HCOOH production can follow two paths via either *COOH or *HCOO, crossed at about −1.3 eV. On A-p, B-p, and C-p, the Δ*G* for HCOOH production is unfavored (about 2.0 eV), which means at least −2.0 V is needed to produce HCOOH on the perfect surfaces. On the contrary, the Δ*G*-determining step on defected surfaces is smaller than 0.5 eV. On A-d and C-d, HER is more favorable than HCOOH and CO. However, HCOOH is most preferred over B-d via the *HCOO pathway, and the product selectivity preference obeys the order of HCOOH > H_2_ > CO, which is exactly consistent with our experiments. Besides, B-d is the most stable termination and should be the dominant site of hydrocerussite. In comparison, the HCOOH activity and selectivity on Pb(111) are lower than the B-d of hydrocerussite, in agreement with the low HCOOH FE of Pb foil about 40% (Supplementary Fig. 4b). The explicit reaction free energy diagrams under 0 V vs. RHE on B-d of hydrocerussite and Pb(111) are displayed in Supplementary Fig. 27. On B-d, HCOOH is more favorable than H_2_ and CO in thermodynamics due to the stronger adsorption of *HCOO than *H and *COOH by 0.61 and 1.24 eV, respectively. On Pb(111), although *HCOO is also more preferred than *H, their small energy difference of only 0.17 eV together with the higher concentration of proton than CO_2_ in an electrolyte, can actually reverse the preference of HCOOH and H_2_. Figure 4b is the two-dimensional map of activity and selectivity of the sites of hydrocerussite, where *G*_ad_(*COOH) is introduced as another dimension and varying independently with *G*_ad_(*HCOO). The map contains four regions, which represent different products. Only B-d shows an obvious HCOOH preference and activity, further confirming B-d as the active site.

To analyze the effect of applied potential on CO_2_RR into HCOOH, the potential-dependent barriers and reaction energies under different experimental potentials are calculated (Fig. 4c). At −0.82 V vs. RHE, the elementary reactions CO_2_(g) + (H^+^ + e^−^) → *HCOO and *HCOO + (H^+^ + e^−^) → HCOOH are both exothermic, with a barrier of 0.82 and 0.57 eV, respectively. In kinetics, CO_2_ protonation is still more difficult than the formation of HCOOH and can be considered as the rate-determining step (RDS). As the potential decreases, the activation energy of CO_2_ protonation becomes lower gradually, which will increase the reaction rate and HCOOH partial current density in the experiment according to the transition state theory. Therefore, we further perform a microkinetic simulation to calculate the charge transfer rate of a single active site (*k*) at different applied potentials. Figure 4d shows the experimental HCOOH partial current density (*j*_exp_) plotted versus our theoretical charge transfer rate. A nice linear correlation has been established, confirming our calculations and analysis above regarding the active site and reaction mechanism.

### Stablity of hydrocerussite

**Fig. 5:**
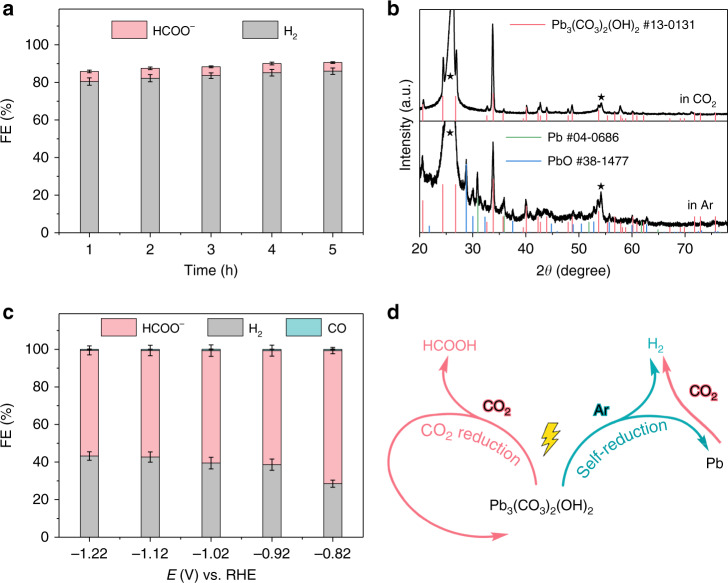
Performances of hydrocerussite without CO_2_ under cathodic potential in NaHCO_3_. **a** FEs of h-PCOH at −0.92 V for 5 h in Ar. **b** Comparison of XRD patterns of h-PCOH after electrolysis under −0.92 V for 5 h in CO_2_ and in Ar. The peaks from the substrate of CP are labelled by black stars. **c** FEs of tested h-PCOH at different potentials in CO_2_. **d** Behaviors of hydrocerussite as the cathodic electrocatalyst. Error bars correspond to the standard error of the mean.

In the whole electrolysis, we attribute the high formate FE and electrochemical stability of hydrocerussite to its dominant CO_2_RR. In this sense, both the electrochemical reduction from hydrocerussite to metal Pb and the competing HER are suppressed. It can be evidenced by the comparison of linear scan voltammetry (LSV) curves in Ar and CO_2_ (Supplementary Fig. 28). All the redox peaks under Ar completely disappear in CO_2_, suggesting the inhibition of Pb reduction under CO_2_ bubbling. Another controlled experiment is to test the stability of h-PCOH at −0.92 V under Ar for 5 h. Different from the results under CO_2_, the FE of hydrogen dramatically rises to over 80%, and the formate FE decreases to around 5% (Fig. 5a and Supplementary Fig. 29). Besides, with the electrolysis time prolonging, the hydrogen FE becomes larger and larger, while the formate FE goes smaller. And no CO can be detected under this condition. It should be noted that the total FE of all the products is obviously smaller than 100%, suggesting that parts of the provided electrons are used to reduce the hydrocerussite. This conjecture can be further confirmed by the XRD pattern of this sample after electrolysis for 5 h. As shown in Fig. 5b, the appearance of XRD peaks from PbO and metal Pb clearly demonstrates the decomposition and reduction of hydrocerussite under this condition, which is in accordance with the Pourbaix diagram of lead in the presence of CO_3_^2−^ ^42^. In addition, when the reduced hydrocerussite is tested in CO_2_ again, obvious decay of formate FE (~60%) can be found just as our expectation (Fig. 5c). From the above results, it is sure that the presence of CO_2_ is of great importance to maintain the stability of hydrocerussite under cathodic conditions. Without CO_2_, hydrocerussite is gradually decomposed and finally reduced to metal Pb, accompanying with enhanced hydrogen evolution and weakened selectivity of formate production (Fig. 5d). In other words, the preference of CO_2_RR on hydrocerussite can effectively suppress the self-reduction of the electrocatalyst and the accompanying enhanced HER.

## Discussion

In summary, we have revealed the dynamic change of TA–Pb complex film as a model pre-catalyst under the conditions of CO_2_RR, and finally unveiled hydrocerussite as the new active species for formate generation. The rapid transformation of TA–Pb to cerussite (t-PCO) and subsequent conversion to hydrocerussite (t-PCOH) are revealed during the electrochemical CO_2_RR conditions. t-PCOH is found to be the active species to produce formate with an optimum FE of 96.4 ± 0.9%. In addition, cerussite nanoparticles are prepared to verify the phase transformation to hydrocerussite. The hydrocerussite nanoplates are also directly fabricated, and perform similar high formate FE and long stability with t-PCOH, further confirming the key role of hydrocerussite. DFT calculations indicate that the hydrocerussite is highly active and selective for formate production. The excellent stability of hydrocerussite originates from its dominance of CO_2_RR, thus the reduction of the electrocatalyst itself and consequent HER is blocked. This work not only opens a facile avenue to improve the CO_2_RR selectivity by suppressing the reduction of the electrocatalysts, but also establishes a guideline for fundamentally understanding the transformation process and active species of metal-complex catalytic materials for cathodic reactions.

## Methods

### Depositing TA–Pb complex on substrates

For a typical synthesis of a single-layer TA–Pb complex, a piece of the substrate was put in a clean and empty 20 mL vessel with the addition of 10 mL 10 mM Pb(NO_3_)_2_. After sufficient adsorption for 15 min, 10 mL 9 mg mL^−1^ tannic acid (TA) solution was quickly added. 2 M NaOH was then added to adjust pH to ~7. Then the vessel was gently shaken and stood at room temperature without disturbance for 6 h. At last, the product was washed with water and dried naturally. To prepare multiple-layer TA-Pb, this procedure was repeated for suitable times. For instance, if this procedure was repeated for three times, the as-prepared products are named as the 3-layer TA–Pb. For environmental concerns, the Pb-containing residual reaction solution was collected and stored in a dedicated container. CP was used as the substrate in electrochemical measurements. Before deposition, CP was cut into rectangular pieces with a size of 1 × 3 cm^2^ and immersed in acetone and water for 15 min in sequence. Rutile TiO_2_ nanorod arrays supporting on F-doped tin oxide (FTO) glass were used as the substrate for TEM observation and ex situ FTIR spectroscopy. FTO glass was cut into a rectangular shape with a size of 1 × 2.5 cm^2^, and sonicated in diluted HCl, acetone, and water for 15 min in sequence. To prepare rutile TiO_2_ nanorod arrays, 15 mL water, 15 mL concentrated HCl, and 0.5 mL tetrabutyl titanate were added to a Teflon-lined stainless-steel autoclave and stirred for 15 min. A piece of clean FTO glass was put into the autoclave with the conductive side facing down. Then the autoclave was sealed and treated at 180 °C for 1 h. After the reactor cooled down naturally, the FTO with the white product was washed with water and alcohol, respectively, and dried naturally. Then the product was calcined in air at 500 °C for 2 h with the heating rate of 5 °C min^−1^ to finally obtain the rutile TiO_2_ nanorod arrays.

### Synthesis of c-PCO nanoparticles

c-PCO nanoparticles were prepared by a simple precipitation method. Briefly, 5 mL 1.2 M Na_2_CO_3_ and 5 mL 1.2 M Pb(OAc)_2_·3H_2_O were mixed and stirred for 5 min. Then 10 mL water was added and stirred for another 1 h at room temperature. The white product was collected by centrifugation and washed with water and ethanol several times, then dried in a vacuum oven for 6 h.

### Synthesis of h-PCOH nanoplates

2.50 g Pb(NO_3_)_2_, 2.25 g urea, 0.075 g polyethylene glycol with the molecular weight of 2000 (PEG-2000), and 200 mL water were added in a flask. The flask was vigorously stirred and refluxed at 105 °C for 5 h. The product was collected by centrifugation and washed with water and ethanol several times, then dried in a vacuum oven for 6 h.

### Characterization

The transmission electron microscopy (TEM) images and high-resolution TEM (HRTEM) images were carried out with a JEOL JEM-2100F microscope. The scanning electron microscopy (SEM) images were taken with a Hitachi S-4800 microscope. The X-ray absorption fine structure (XAFS) were performed at the 1W1B beamline of Beijing Synchrotron Radiation Facility (BSRF). And the XAFS spectra were analyzed with the ATHENA software package^43^. The *k*-weighting was set to 1 for the Fourier transforms. Fourier transforms of *χ*(*k*) were performed in the k-range of 2–8 Å^−1^ with the Hanning window function. All the EXAFS spectra were without phase correction. The gaseous products of electrochemical CO_2_RR were detected by a GC (Shimadzu GC-2010). And the formate was measured by a 400 MHz NMR spectrometer (Bruker AVANCE III HD). The determination of formate was based on the internal standard method by using 100 ppm dimethylsulfoxide (DMSO) as the internal standard substance. The XRD patterns were recorded on a Bruker D8-Focus diffraction system with a Cu *Kα* source (*λ* = 1.54056 Å). The ICP-MS was collected on an Agilent 7700X instrument. The XPS were carried out with a Thermo ESCALAB 250XI spectrometer. All the peaks were calibrated with C 1s spectrum at the binding energy of 284.8 eV. The Raman spectra were recorded on a Renishaw inVia reflex Raman microscope under an excitation of 532 nm laser with the power of 20 mW. The ex situ FTIR were taken with a Bruker ALPHA-T FTIR spectrometer.

### Electrochemical measurements

All the electrochemical measurements were taken with a CHI 650e electrochemical workstation (CH Instruments, Austin, TX). A typical three-electrode system was employed in a home-made H-type electrolytic cell. Generally, 0.5 M NaHCO_3_ was used as the electrolyte. Only with ^13^CO_2_ as the feedstock, 0.5 M Na_2_SO_4_ was used as the electrolyte to eliminate the effect of carbonate species from NaHCO_3_. Before the measurements, Ar or CO_2_ was bubbled to the system for over 30 min to remove the dissolved oxygen and continuously bubbled throughout the measurements. TA–Pb supported on CP and Pb foil were used as a working electrode directly. For the powder samples like c-PCO and h-PCOH, 5 mg sample was first dispersed in 1 mL ethanol with the addition of 20 μL 5% Nafion 117 solution. After sufficient sonification, all the 1 mL homogeneous ink solution was carefully dropped on a piece of CP with the exposed area of 1 cm^2^ by epoxy coating, making the mass loading to be 5 mg cm^−2^. Saturated calomel electrode (SCE) and Pt sheet were used as the reference electrode and counter electrode, respectively. All the potentials mentioned in this work were against the RHE without additional description. The scan rate for all the voltammetry was 10 mV s^−1^. Before all CO_2_RR measurements, each sample was tested at −0.92 V for 30 min to achieve the steady state. For potential-dependent FE measurements, the sample was maintained at the specific potential for another 30 min to accumulate enough products. For environmental concerns, we analyzed the Pb content in the electrolyte after tests by ICP-MS. The result showed that only 4.418 μg L^−1^ Pb was detected, which was obviously lower than the Chinese Standards for Drinking Water Quality (GB 5749-2006) of 0.01 mg L^−1^ for Pb, indicating that the use of hydrocerussite as electrocatalyst for CO_2_RR was eco-friendly.

### Calculation of TOF

The calculation of TOF is based on Eq. (1) as follows.

1 $${\mathrm{TOF}} = \frac{{{\it{n}}_{\mathrm{f}}}}{{{\it{n}}_{\mathrm{c}} \times {\it{t}}}},$$

where ${\it{n}}_{\mathrm{f}}$ is the amount of the generated formate, ${\it{n}}_{\it{c}}$ is the amount of the active catalyst site, and *t* is the duration time. In this work, we assume all the Pb sites are active for the reaction. The generated formate is calculated according to Eq. (2).

2 $${\it{n}}_{\mathrm{f}} = \frac{{{\it{Q}} \times {\mathrm{FE}}_{\mathrm{f}}}}{{2 \times {\it{F}}}},$$

where ${\it{Q}}$ is the integrated coulombs from the *I-t* curve of TA-Pb, FE_f_ is the FE of formate, and *F* is the Faradaic constant.

### Electrochemical in situ spectroscopy

In situ ATR-FTIR spectra were obtained on a Nicolet 6700 FTIR spectrometer with silicon as the prismatic window. A thin layer of gold film was chemically deposited on the surface of the silicon prismatic prior to each experiment. Then 20 μL sample ink was carefully dropped on the surface of the gold film, together served as the working electrode. Pt sheet and Ag/AgCl were used as the counter electrode and the reference electrode, respectively. 0.5 M NaHCO_3_ was employed as the electrolyte. CO_2_ or argon was bubbling to the electrolyte in advance and continuously bubbled throughout the experiment. The in situ Raman spectroscopy of the decomposition of TA–Pb was carried out on the same Raman microscope mentioned before at −1.22 V vs. RHE in 0.5 M NaHCO_3_ with CO_2_ bubbling. The electrolytic cell was homemade by Teflon with a piece of round quartz glass as the cover to protect the objective of the microscope. A piece of TA–Pb supported on CP was inserted through the wall of the cell to keep the plane of the working electrode perpendicular to the incident laser. Pt wire as the counter electrode was rolled to a circle around the working electrode. SCE was used as a reference electrode. Quasi-in-situ XAFS spectra were acquired by measuring the electrochemical performances of the sample just near the synchrotron radiation facility, then the tested sample was immediately delivered to the facility for XAFS measurements.

### Computational details

DFT calculations were performed with the Vienna Ab initio Simulation Package^44,45^. The revised Perdew-Burke-Ernzerhof (rPBE)^46^ exchange-correlation functional was adopted as the level of generalized gradient approximation (GGA). The atomic valence orbitals were described by plane-wave basis sets with the kinetic cutoff energies of 400 eV. A Gaussian smearing with a width of 0.2 eV was used. All the total energy calculations were converged at 0.05 eV/Å. The hydrocerussite (0001) surface with a vacuum of 20 Å was used to model experimental hydrocerussite samples. Six layers were contained in the slab, with the top three relaxed and the rest fixed. A (4 × 4) Pb(111) slab with 4 layers was used to model Pb foil, where the top two were allowed to be relaxed. To calculate the protonation barriers, a layer of H_2_O with a density of ~1 g cm^−2^ was introduced upon the surface of catalysts.

### Reaction phase diagram

All the adsorption free energies of intermediates were first calculated, referenced to CO(g), H_2_(g), and H_2_O(g). All the adsorption free energies could be correlated with that of *HCOO, in the format of *G*_ad_(*X) = *a* × *G*_ad_(*HCOO) + *b*, where *G*_ad_(*X) was the adsorption free energy of intermediate X and *G*_ad_(*HCOO) was that of *HCOO, used as descriptor herein. Then, the reaction free energy (Δ*G*) of all involved elementary steps, such as *COOH + (H^+^ + e^−^) → HCOOH, can be calculated by Δ*G* = *G*(HCOOH) − *G*(H^+^ + e^−^) − *G*_ad_(*COOH), where *G*(HCOOH) was the chemical potential of formic acid and *G*(H^+^ + e^−^) was referred to 1/2 H_2_ under *U* = 0 V vs. RHE^47^, respectively, referenced to the same criterion with adsorption energies. Obviously, *G*(HCOOH) and *G*(H^+^ + e^−^) were constants, and *G*_ad_(COOH*) was linearly correlated with *G*_ad_(*HCOO). Therefore, the reaction free energy as a linear function of *G*_ad_(*HCOO) could be obtained (Fig. 4a), which was defined as the reaction phase diagram in our previous report^48^. Besides, a two-dimensional map of activity and selectivity (Fig. 4b) could be obtained by making *G*_ad_(*COOH) vary independently with *G*_ad_(*HCOO) and analytically calculating the corresponding reaction free energy at every pair of *G*_ad_(*COOH) and *G*_ad_(*HCOO), which can give a more accurate description on the activity and selectivity.

### Potential-dependent barrier and reaction energy

The potential effect on thermodynamic reaction free energy was considered by changing the chemical potential of (H^+^ + e^−^) by –*eU*, as suggested in the computational hydrogen electrode^47^ approximation. The potential-dependent kinetic barriers of electrochemical reaction (*A + H^+^ + e^−^ → *AH) were calculated by using an equivalent analogous non-electrochemical reaction (*A + *H → *AH) combined with Marcus theory, an effective method developed by Janik et al.^49,50^.

### Microkinetics simulations

The rate of CO_2_ protonation is considered as the total reaction rate since it is the RDS. The rate constant (*a*) was determined by:

3 $${\it{a}} = {\it{A}}{\mathrm{e}}^{\frac{{ - {\it{G}}_{\mathrm{a}}}}{{{\it{k}}_{\it{b}}{\it{T}}}}},$$

where *G*_a_ is the activation free energy; *k*_*b*_ is the Boltzmann constant and *T* is temperature. *A* is the prefactor and calculated by:

4 $${\it{A}} = \frac{{{\it{k}}_{\it{b}}{\it{T}}}}{{\it{h}}}\frac{{{\it{Q}}_{{\mathrm{ts}}}}}{{\it{Q}}},$$

where *h* is the Planck constant; *Q*_ts_ and *Q* refer the partition functions of the transition and initial states, respectively. *A* was approximated to be 10^13^ s^−1^ for surface reactions in this work. The forward rate of CO_2_ protonation [CO_2_(g) + (H^+^ + e^−^) + * → *HCOO] was calculated by:

5 $${\it{r}} = {\it{ac}}\left( {\mathrm{CO}_2} \right){\it{c}}\left( {\mathrm{H}^ + } \right)\theta \ast$$

6 $$= A\;e^{^{\frac{{ - {\it{G}}_{\it{a}}}}{{{\it{k}}_{\it{b}}{\it{T}}}}}}\left[ {{\it{c}}\left( {\mathrm{CO}_2} \right){\it{c}}\left( {\mathrm{H}^ + } \right)\theta \ast } \right],$$

where *c*(CO_2_), *c*(H^+^) and θ* are the concentration of CO_2_ and proton in electrolyte and coverage of the free active site, respectively. The charge transfer rate on a single active site (*k*) is:

7 $${\it{k}} = {\it{rzF}}$$

8 $$= A\;e^{\frac{{ - {\it{G}}_{\it{a}}}}{{{\it{k}}_{\it{b}}{\it{T}}}}}\left[ {{\it{c}}\left( {\mathrm{CO}_2} \right){\it{c}}\left( {\mathrm{H}^ + } \right)\theta \ast } \right]{\it{zF}},$$

where *z* is the total number of electrons transferred in the overall reaction, *F* = 1.6 × 10^−19^.

In principle, *c*(CO_2_), *c*(H^+^) and θ* are constant at different potentials, hence the term [*c*(CO_2_)*c*(H^+^)θ*] is assumed to be 1 in this study. By this approximation, the charge transfer rate *k* under different potentials can be estimated.

## Supplementary information

Supplementary Information

Peer Review File

## Data Availability

The source data underlying Figs. 1–5 are provided as a Source Data file. The data that support other plots within this paper are available from the corresponding author upon reasonable request.

## Source data

Source Data
