# Supplementary material for: Unveiling hydrocerussite as an electrochemically stable active phase for efficient carbon dioxide electroreduction to formate
Source: Nat Commun. 2020 Jul 8;11:3415. doi: 10.1038/s41467-020-17120-9 (PMC7343827; doi:10.1038/s41467-020-17120-9)
Supplement: Supplementary file 1 — Supplementary Information [file 41467_2020_17120_MOESM1_ESM.pdf]

Supplementary Information

Unveiling hydrocerussite as an electrochemically stable active phase for efficient carbon dioxide electroreduction to formate

Shi et al.

Supplementary Figures

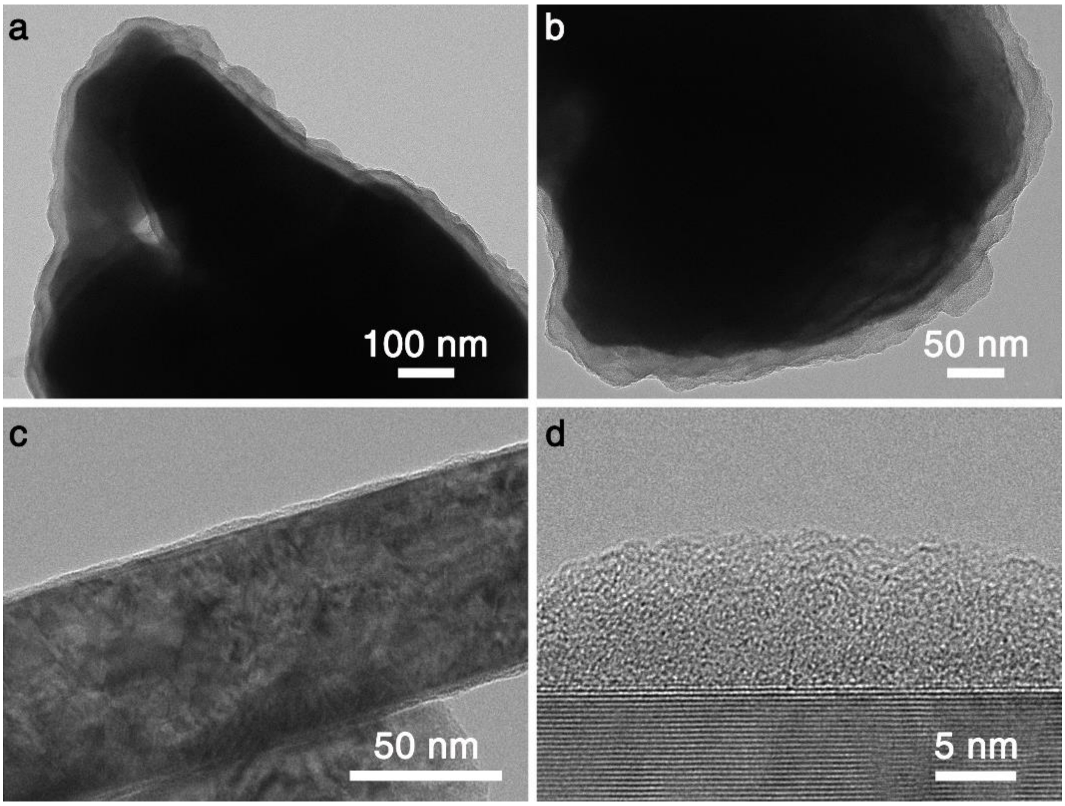

Supplementary Figure 1. TEM images of single-layer TA-Pb. **a** and **b** TEM images of single-layer TA-Pb with CP as the substrate. **c** TEM image and **d** HRTEM image of single-layer TA-Pb with rutile TiO_2 as substrate.

Supplementary Note 1. TA-Pb supported on CP is sonicated into pieces for the observation of TEM. As shown in Supplementary Figure 1a-b, the coating of single-layer TA-Pb on CP is dense and homogeneous around the carbon fragment. However, it is difficult to clearly distinguish the boundary of the complex and the substrate because of the amorphous feature of them both (inset in Figure 1a). To clearly study the boundary and the evolution of TA-Pb during electrolysis, well-crystalline TiO_2 nanorods with a diameter of ~100 nm are chosen as the supported material for the observation of TEM (Supplementary Figure 1c). The previous report has shown that the thickness of tannic complexes may vary with the change of the substrate material.[1] In our case, we can find that the thickness of TA-Pb on TiO_2 prepared through the same conditions decreases to ~8 nm with a clear boundary (Supplementary Figure 1d). So the TEM images of the TiO_2@TA-Pb can only be used to approximately reflect the trend of film evolution during electrolysis rather than the absolute state of CP@TA-Pb.

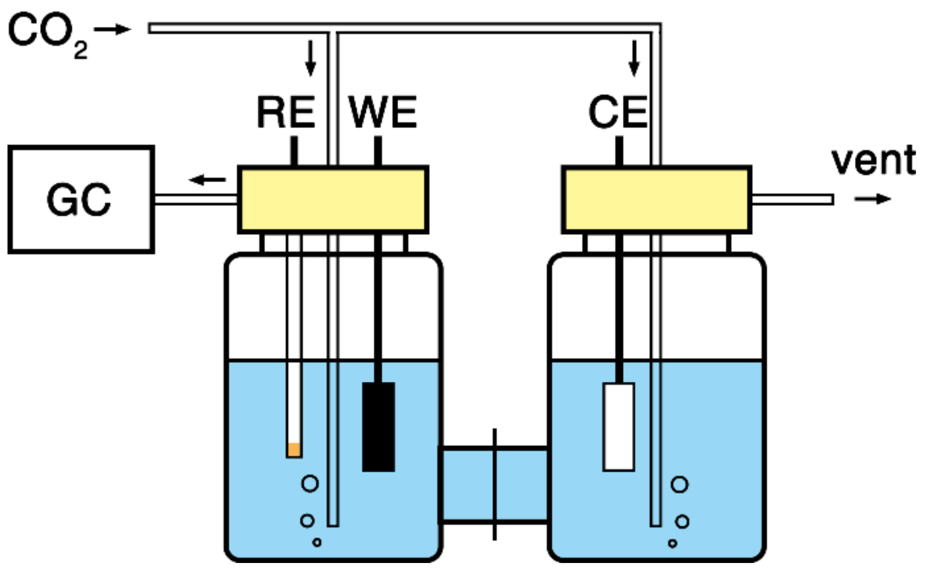

Supplementary Figure 2. Illustration of the home-made electrochemical CO_2RR set up used in this work.

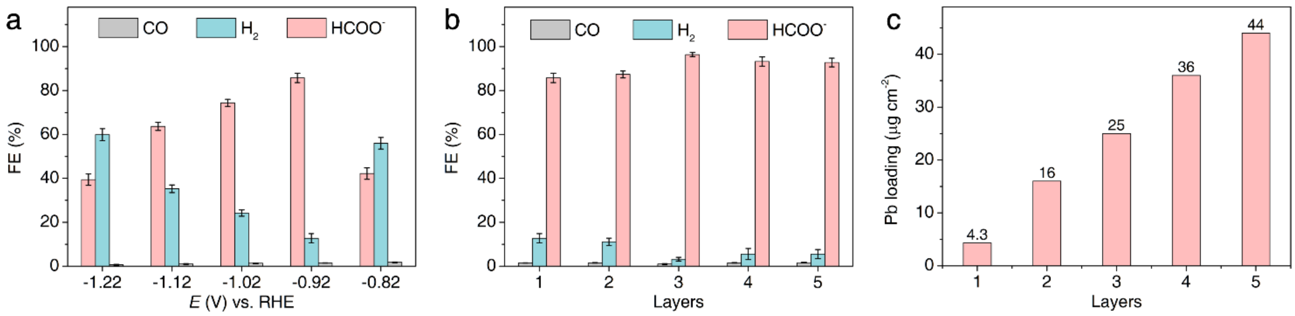

Supplementary Figure 3. Layer optimization of TA-Pb pre-catalyst. **a** FEs of single-layer TA-Pb at different potentials. **b** FEs of different layers of TA-Pb at the optimum potential of -0.92 V. **c** Pb loading of TA-Pb with different layers. Error bars correspond to the standard error of the mean.

Supplementary Note 2. The preparation of single-layer TA-Pb is described in the experimental section. And the multiple-layer TA-Pb is fabricated by repeating the above procedure for specific times. It can be seen that -0.92 V is the optimum potential for formate generation among these tested potentials. And when TA-Pb with different layers is tested at this optimum potential, 3-layer TA-Pb shows the highest FE of formate. It can be directly found that Pb loading increases with the layers, thus affecting the formate FE.

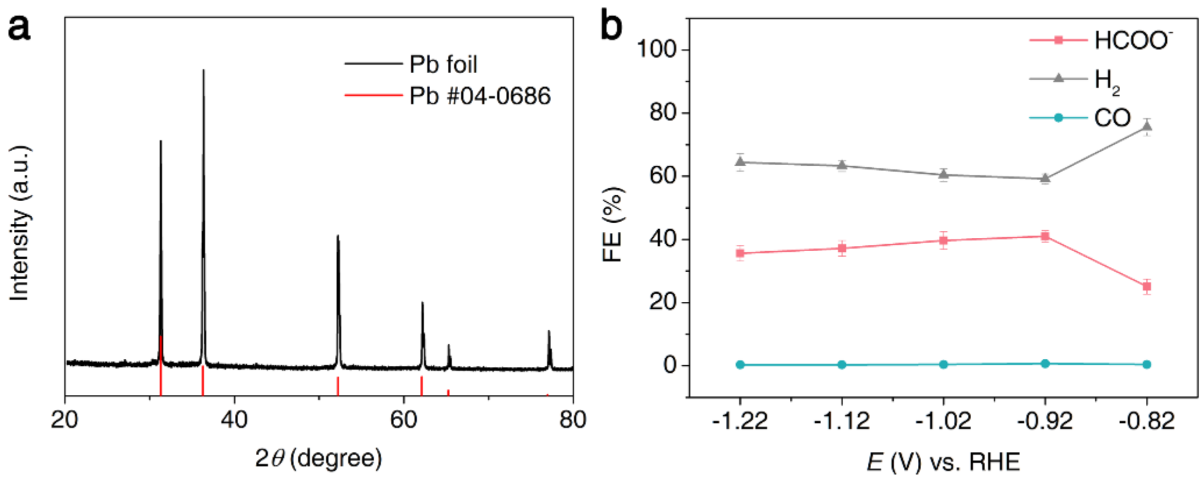

Supplementary Figure 4. Characterizations of Pb foil. **a** XRD patterns of Pb foil. **b** FEs of Pb foil at different potentials. Error bars correspond to the standard error of the mean.

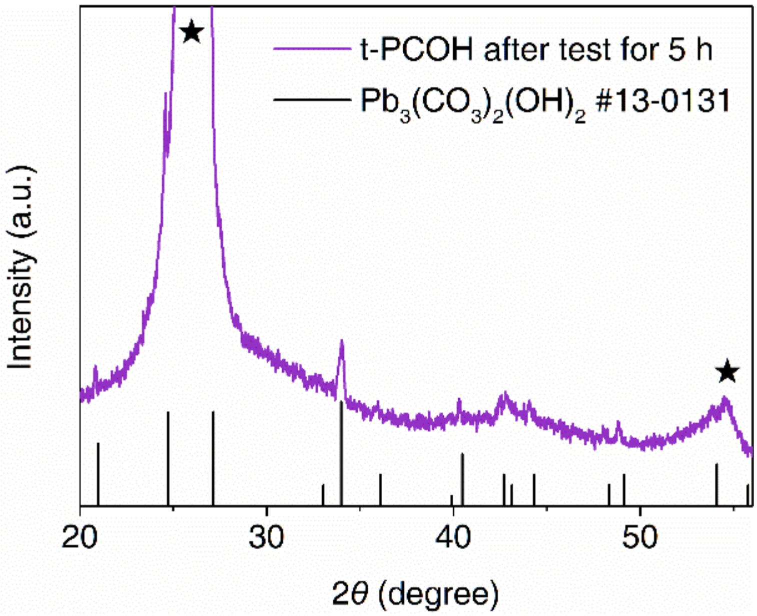

Supplementary Figure 5. XRD pattern of t-PCOH after the test at -0.92 V for 5 h. The peaks from the substrate are labelled by black stars. The corresponding XRD matches well with hydrocerussite without any impurity, indicating no Pb(0) is formed during the process.

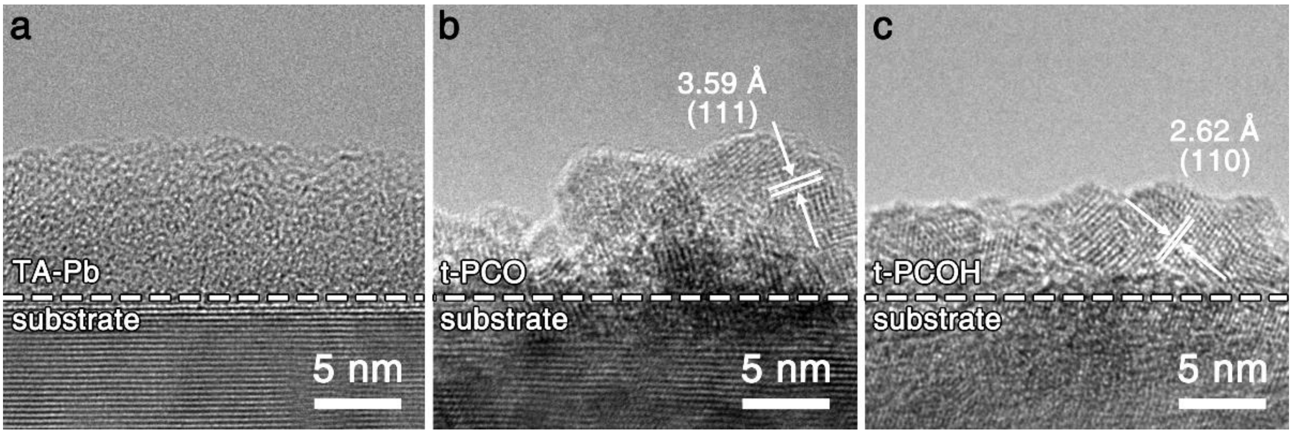

Supplementary Figure 6. HRTEM images of **a** TA-Pb, **b** t-PCO, and **c** t-PCOH with rutile TiO_2 as the substrate.

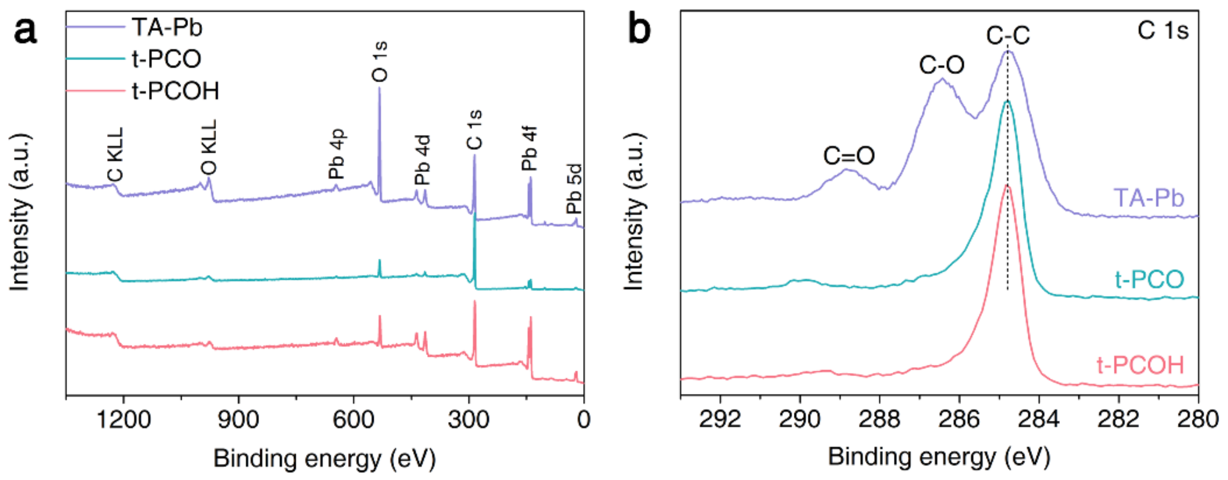

Supplementary Figure 7. XPS spectra of TA-Pb, t-PCO and t-PCOH. **a** Survey spectra. **b** C 1s spectra.

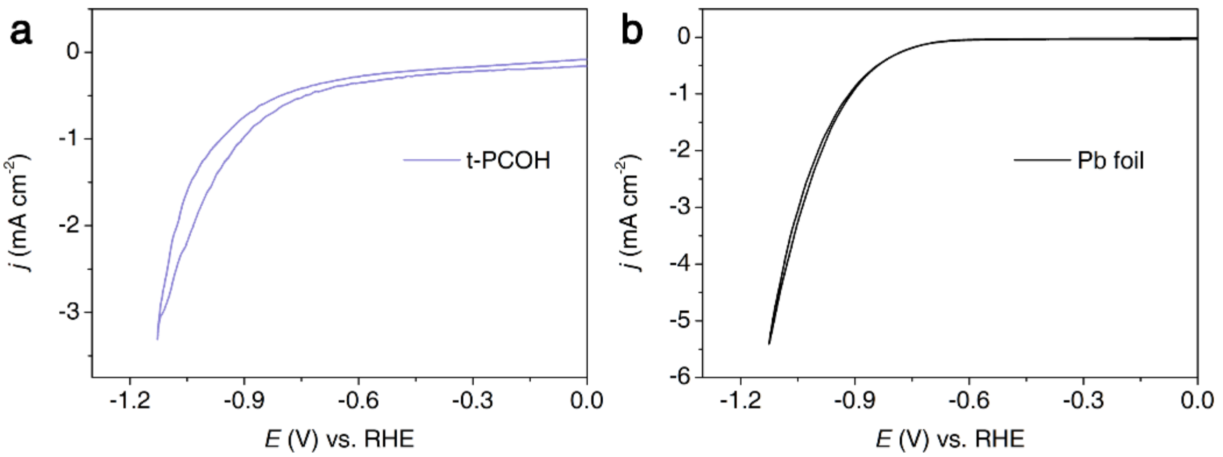

Supplementary Figure 8. CV curves of samples tested at the presence of CO_2. **a** t-PCOH. **b** Pb foil. No redox peak can be found in the whole voltammogram of t-PCOH, indicating that the valence of Pb is maintained.

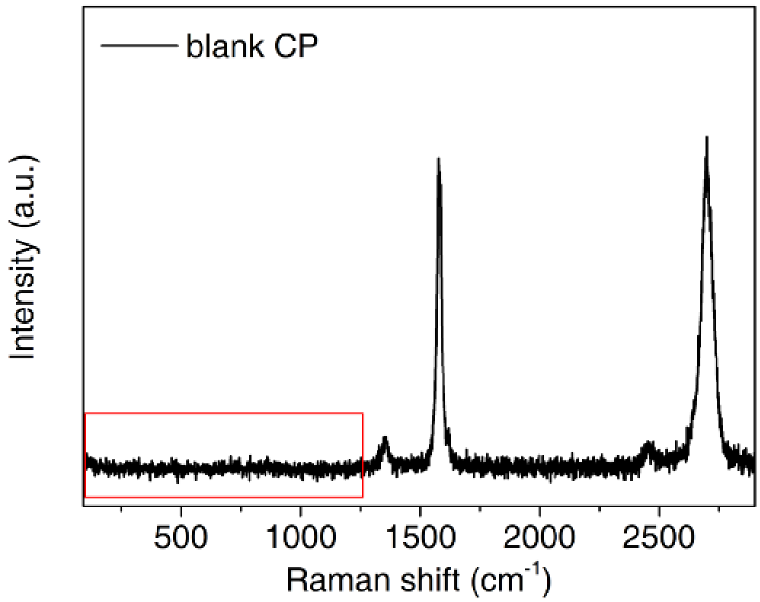

Supplementary Figure 9. *Ex situ* Raman spectra of CP. No peak appears in the range of 100-1200 cm^{-1} for CP, qualifying CP to be an adequate substrate for *in situ* Raman spectroscopy.

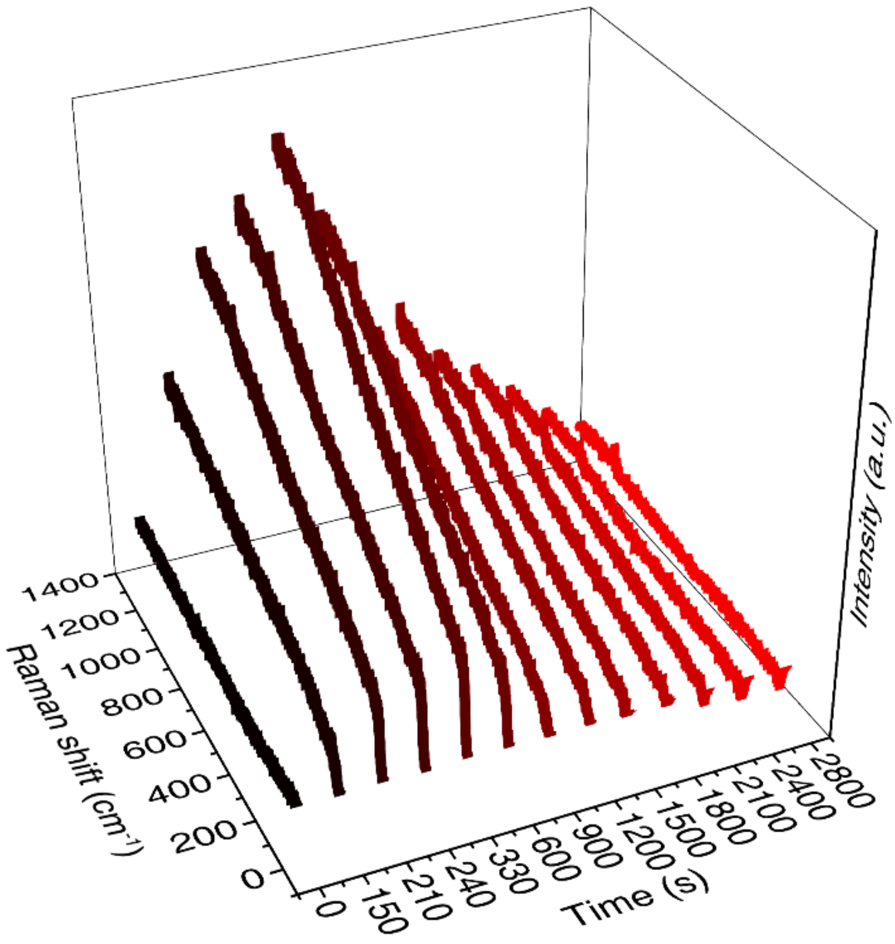

Supplementary Figure 10. Three-dimensional time-dependent *in situ* Raman spectra of TA-Pb.

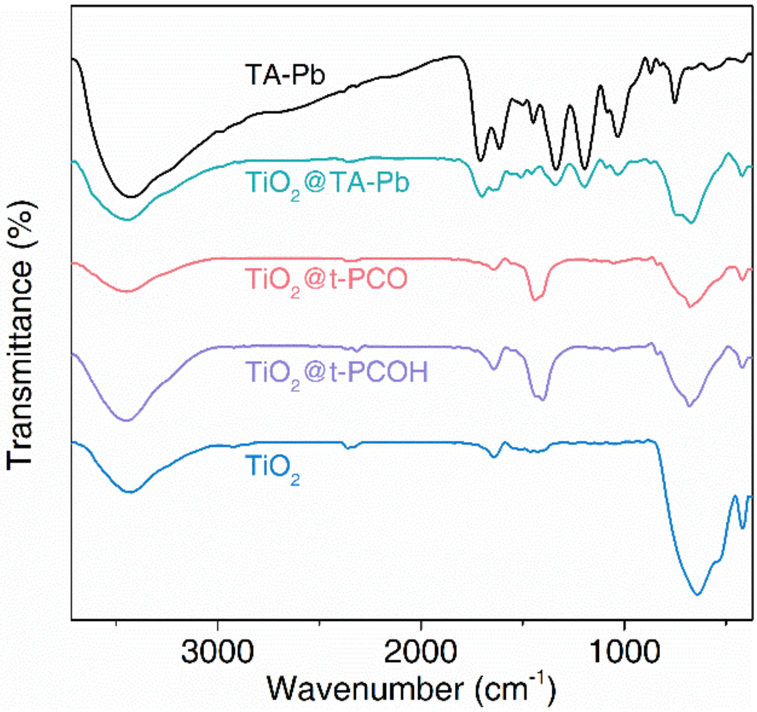

Supplementary Figure 11. *Ex situ* FTIR spectra of TA-Pb at different transformation stages with TiO_2 as substrate. All the peaks attributed to TA-Pb complex film disappear in t-PCO and t-PCOH, suggesting the dissociation of the complex.

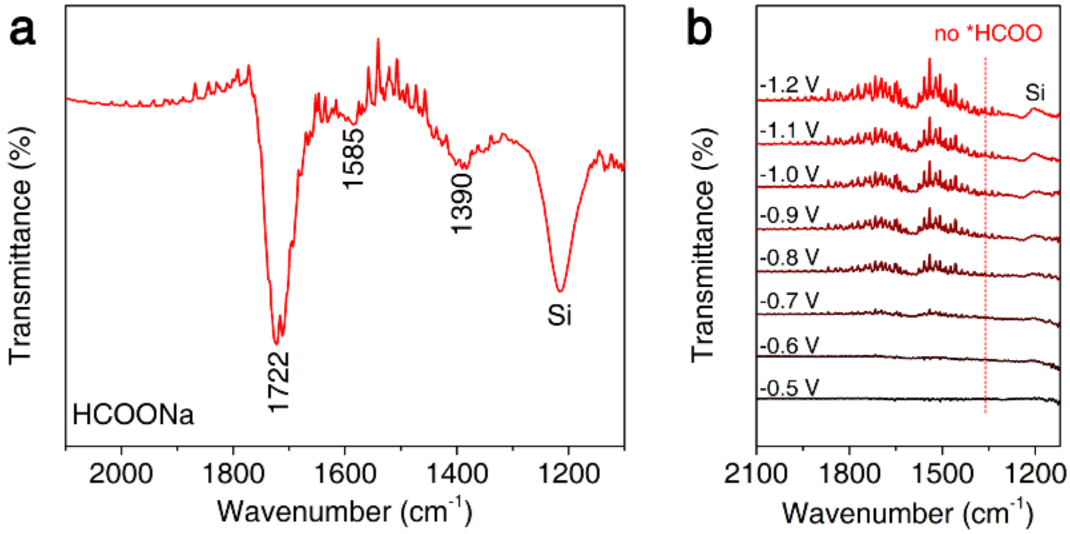

Supplementary Figure 12. Additional ATR-FTIR spectra. **a** ATR-FTIR spectrum of HCOONa aqueous solution obtained under the same condition with Figure 2h. **b** Potential-dependent *in situ* ATR-FTIR spectra of PCOH with argon bubbling in 0.5 M $NaHCO_3$.

Supplementary Note 3. In Supplementary Figure 12a, the bands at 1722 and 1585 cm^{-1} can be attributed to C=O vibration of formate, and the band at 1390 cm^{-1} can be assigned to the vibration of C-O in formate.[2] It can be seen that the strongest C=O band at 1722 cm^{-1} is the characteristic feature of formate. However, no corresponding band can be found in Figure 2h, indicating that the bands of 1360 cm^{-1} in Figure 2h is attributed to the *HCOO instead of the generated formate.

When replacing CO_2 by Ar, no bands represented HCOO* can be found throughout the potential, even with the same 0.5 M $NaHCO_3$ as the electrolyte (Supplementary Figure 12b). The result suggests that the gaseous CO_2 is the primary source for formate generation on PCOH rather than bicarbonates in the electrolyte.

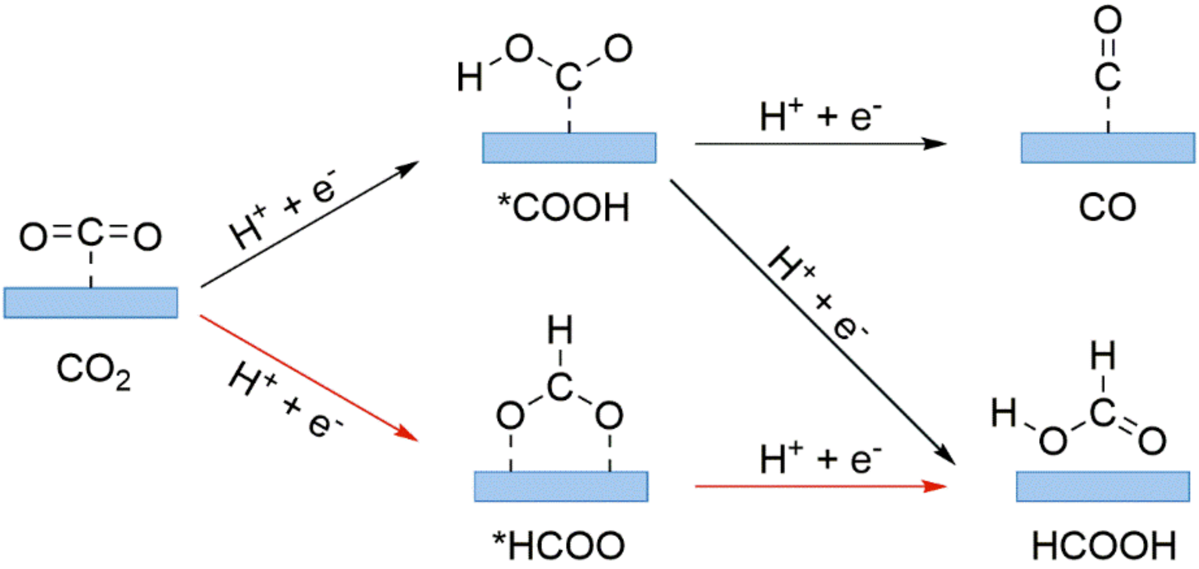

Supplementary Figure 13. Reaction paths for formate generation. The dominant path in our work is labelled by red color.

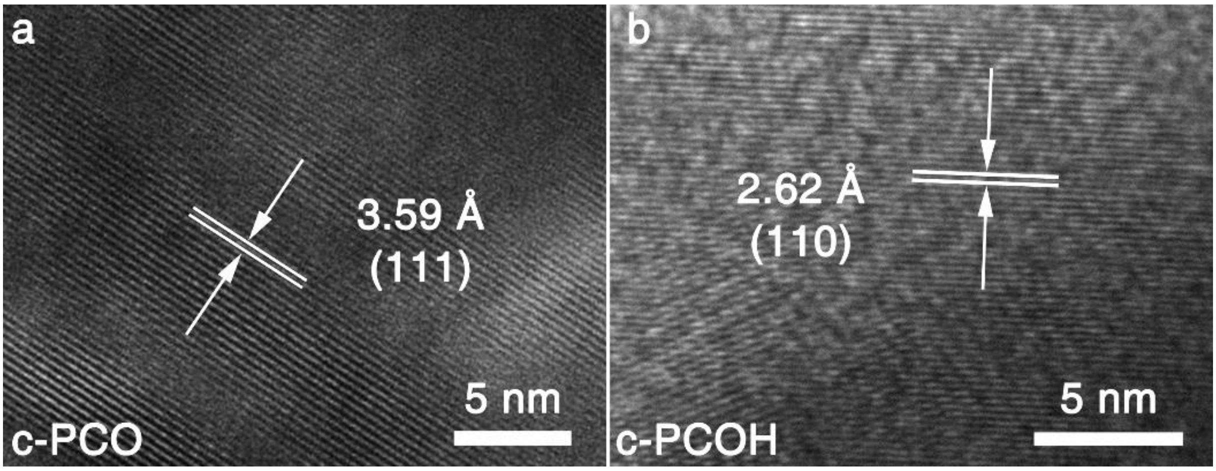

Supplementary Figure 14. HRTEM images of c-PCO and c-PCOH. **a** c-PCO. **b** c-PCOH.

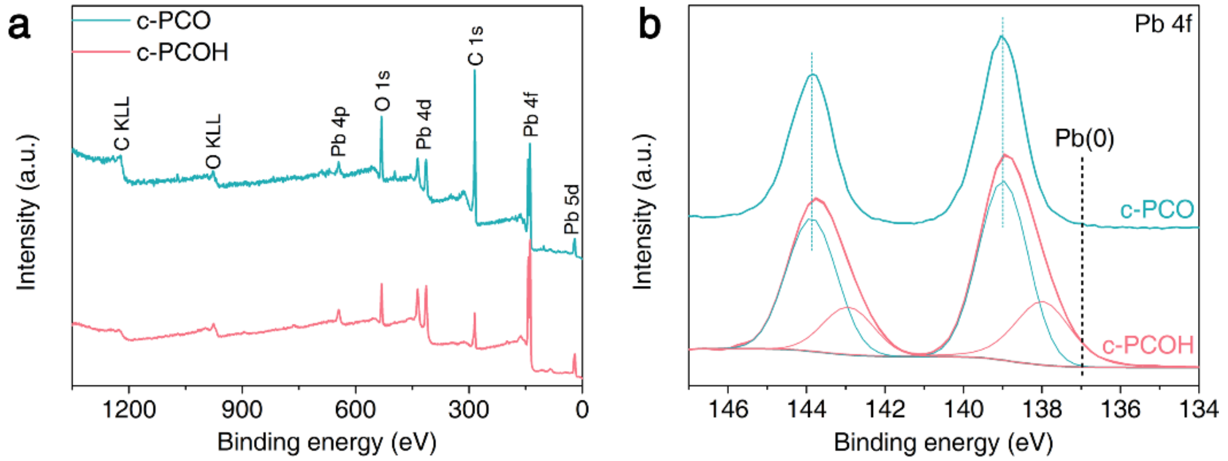

Supplementary Figure 15. XPS spectra of c-PCO and c-PCOH. **a** Survey spectra. **b** Pb 4f spectra. After the transformation, the composition of c-PCOH does not change. In the Pb 4f XPS spectra, peaks at 139.0 and 143.8 eV are associated with $PbCO_3$. And peaks appeared at 138.0 and 142.9 eV can be assigned to Pb-OH.[3] No peak attributed to Pb(0) is found at the binding energy of ~137.0 eV.

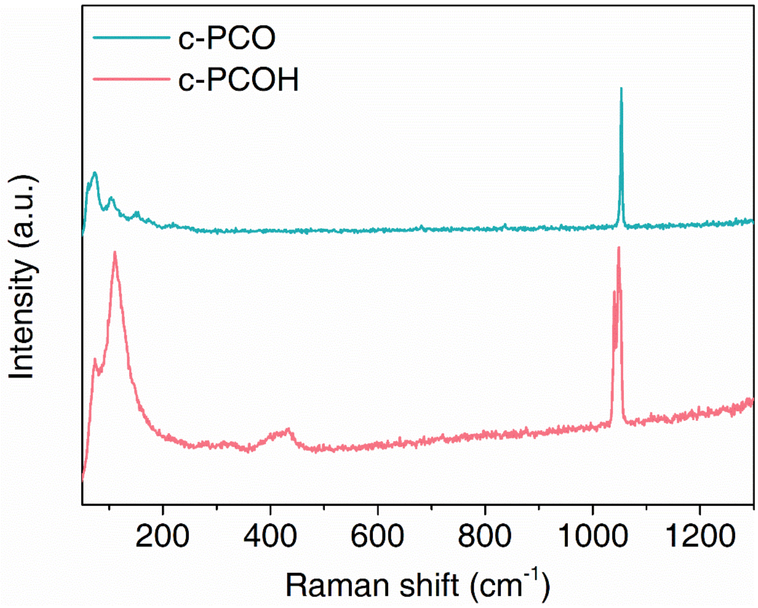

Supplementary Figure 16. *Ex situ* Raman spectra of c-PCO and c-PCOH.

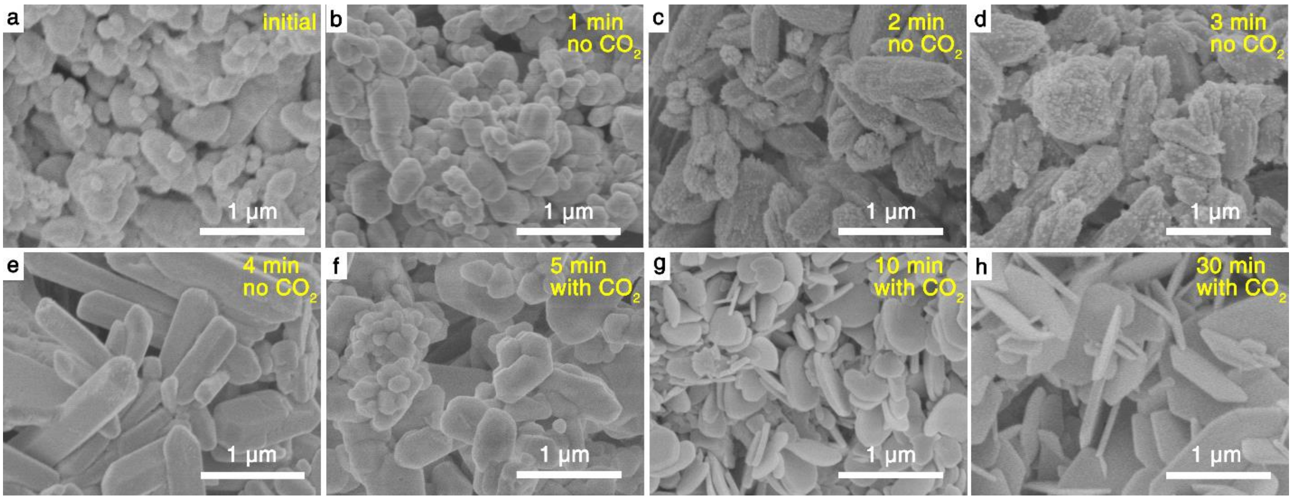

Supplementary Figure 17. SEM images of c-PCO as pre-catalyst tested at -0.92 V for different time.

Supplementary Note 4. Without CO_2 bubbling, it can be seen that the sample transforms from irregular nanoparticles to nanoprisms (in the first 4 min, a-e). Only with CO_2 bubbling, can the nanoprisms gradually shorten and finally form the nanosheets (5-30 min, f-h).

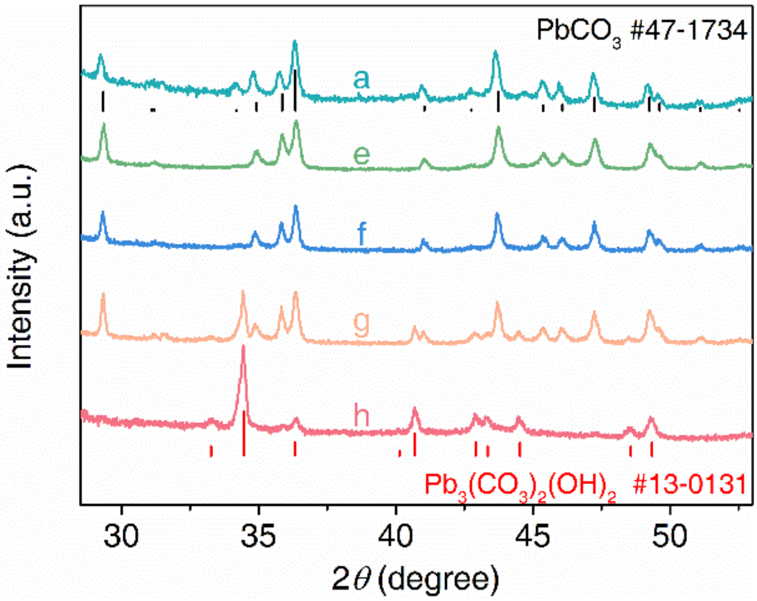

Supplementary Figure 18. XRD patterns of c-PCO as the pre-catalyst tested at -0.92 V for different time corresponding to Supplementary Figure 17. The letters in this figure are in accordance with Supplementary Figure 17, which represents, a, initial; e, 4 min without CO_2; f, 5 min with CO_2; g, 10 min with CO_2; and h, 30 min with CO_2.

Supplementary Note 5. Combining with Supplementary Figure 17, we can find that without CO_2, although huge shape change happens to the sample, the crystalline structure of the sample does not change. After CO_2 bubbling, the cerussite gradually converts to hydrocerussite. And the formation of hydrocerussite begins at about 10 min with CO_2 bubbling. After electrolyzing under CO_2 for 30 min, all the cerussite completely converts to hydrocerussite.

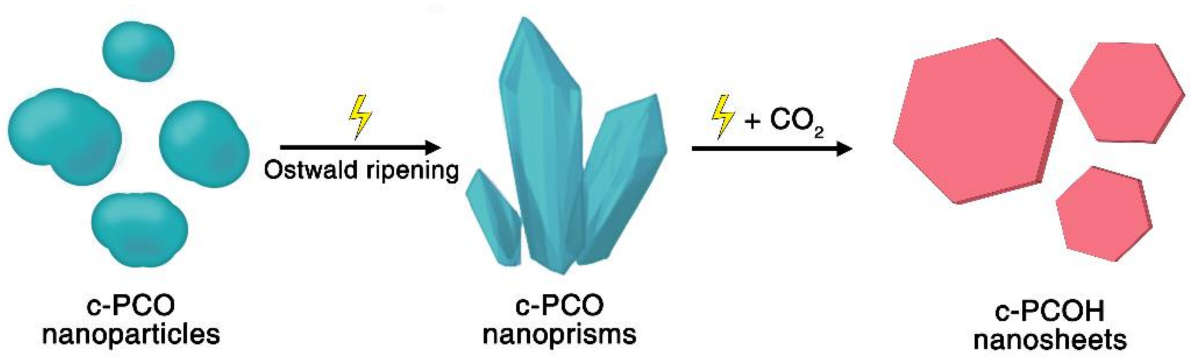

Supplementary Figure 19. Schematic illustration of the transformation mechanism from c-PCO to c-PCOH.

Supplementary Note 6. As shown in Supplementary Figure 17-18, the transformation intermediates are captured at different reaction stages and characterized by SEM and XRD. In this system, only applied potential and bubbled CO_2 may influence the transformation process. So we have separately discussed the effects of both factors. It can be found that in the absence of CO_2, the c-PCO nanoparticles can gradually convert to c-PCO nanoprisms under -0.92 V (Supplementary Figure 17a-e). Only under the stimulation of both CO_2 bubbling and applied potential, can the c-PCO nanoprisms further transform into c-PCOH nanosheets (Supplementary Figure 17f-h). Briefly, the applied cathodic potential is necessary all the time. But the second transformation from c-PCO nanoprisms to c-PCOH nanosheets cannot be achieved without CO_2.

Since valences of Pb are the same in cerussite and hydrocerussite, it is suggested that the function of the applied potential is to increase the local pH *via* HER. Specially, in 0.5 M $NaHCO_3$, the value of pH is about 8.3. So HER happens as follow equation (1).

$$2H_2O + 2e^- \rightarrow H_2 + 2OH^- \quad (1)$$

As cerussite is soluble in strong alkaline media, the increased local pH can make a small amount of c-PCO dissolved and recrystallized *via* the Ostwald ripening process.[4, 5] As a result, the c-PCO nanoparticles gradually convert to c-PCO nanoprisms.

With CO_2 bubbling into the electrolyte, the pH of the system decreases to 7.4. At this moment, HER together with CO_2RR can continuously increase the local pH (2).

$$CO_2 + 2H_2O + 2e^- \rightarrow HCOO^- + 2OH^- \quad (2)$$

We suppose that CO_2 may etch the nanoprisms along their c-axis, accompanied by the insertion of OH^- into the catalyst. As a result, the c-PCOH nanosheets are formed with (0001) as the dominant exposed facet. The proposed reaction equation from c-PCO nanoprisms to c-PCOH nanosheets are suggested as follows (3).

$$3PbCO_3 + 4OH^- + CO_2 \rightarrow Pb_3(CO_3)_2(OH)_2 + 2CO_3^{2-} + H_2O \quad (3)$$

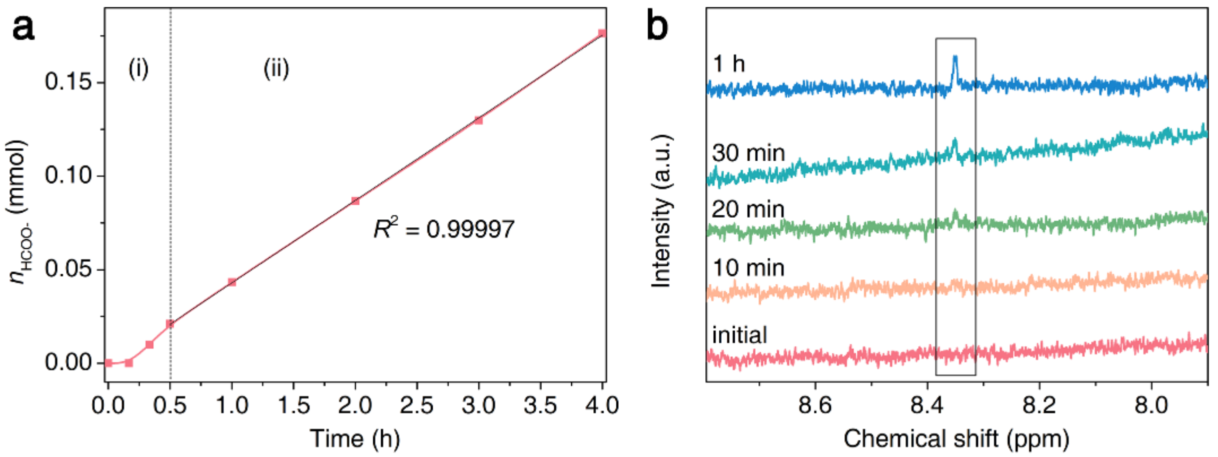

Supplementary Figure 20. Formate production of c-PCO and c-PCOH under -0.92 V at different time. **a** The production of formate. **b** Corresponding ^{1}H-NMR spectra of generated formate. The characteristic bands of generated formate at ~8.35 ppm are labeled by a black box.

Supplementary Note 7. The formate production of c-PCO in Supplementary Figure 20a can be divided into two parts based on the generation rate. At the first half an hour (stage i), the generation rate of formate increases gradually. And even no formate is generated at the first 10 min. This period is in accordance with the transformation from c-PCO to c-PCOH discussed above (Supplementary Figure 19), suggesting that cerussite is inactive for formate generation. After 30 min (stage ii), the generation rate of formate becomes constant, indicating that the transformation is complete and c-PCOH is responsible and stable for formate generation.

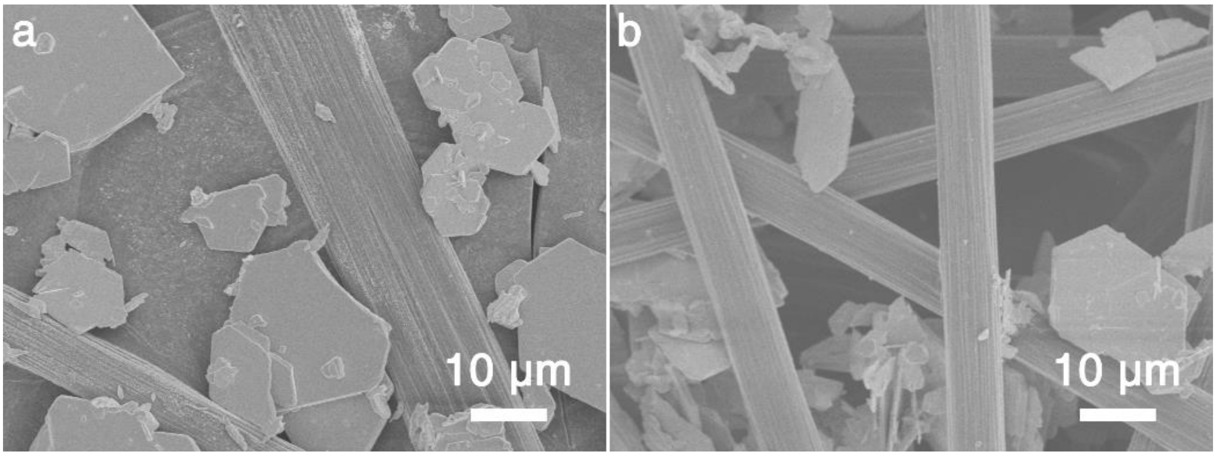

Supplementary Figure 21. SEM images of h-PCOH supported on CP before and after CO_2RR test for 5 h. h-PCOH with the size of several mircometers cannot cover CP as the substrate very well, leading to a relatively low formate FE. Besides, no obvious change is found before and after the electrochemical test, showing the high stability of h-PCOH.

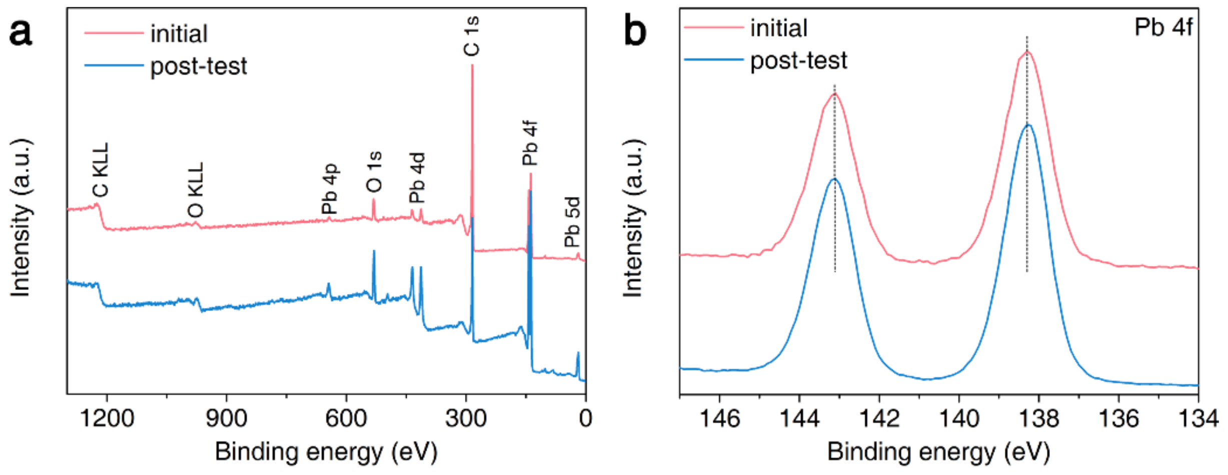

Supplementary Figure 22. XPS spectra of h-PCOH before and after the CO_2RR test for 5 h. **a** Survey spectra. **b** Pb 4f spectra.

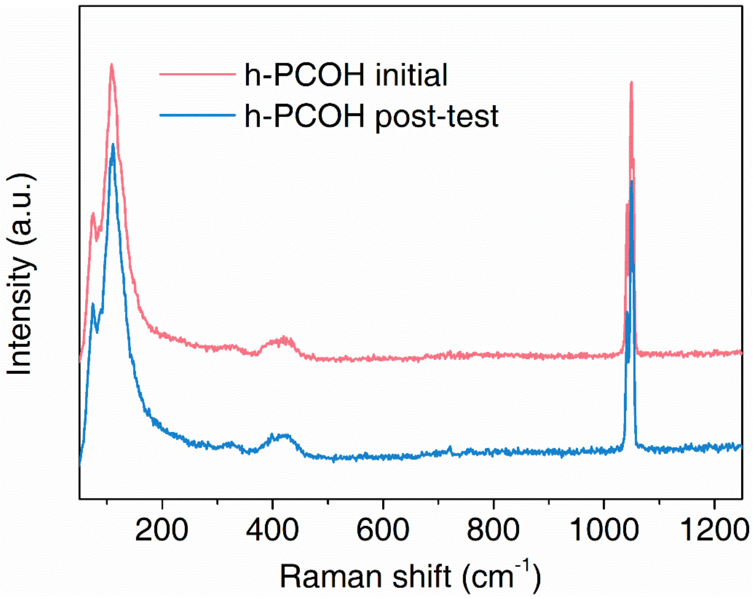

Supplementary Figure 23. *Ex situ* Raman spectra of h-PCOH before and after the CO_2RR test for 5 h.

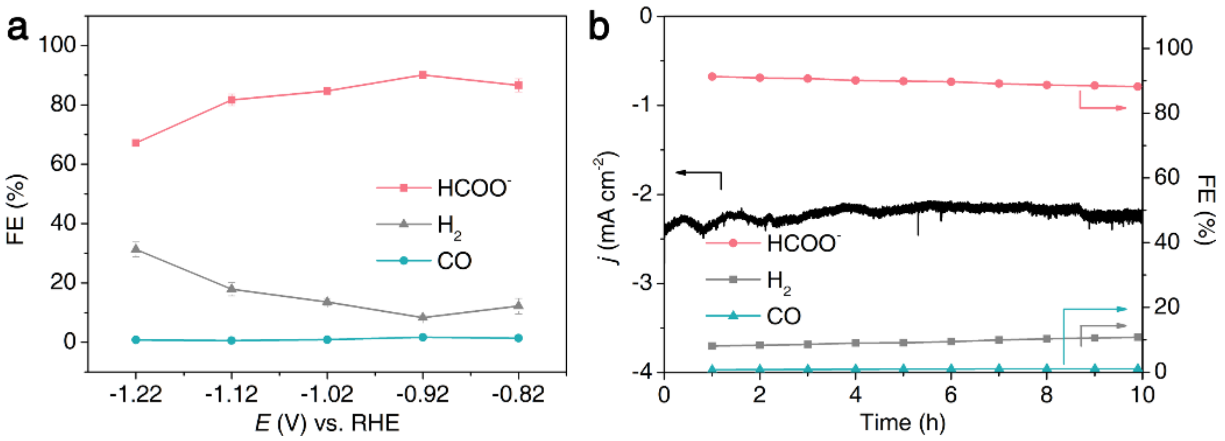

Supplementary Figure 24. Electrochemical CO_2RR of h-PCOH. **a** Formate FEs of h-PCOH. **b** *j*-*t* curves and corresponding formate FEs of h-PCOH at -0.92 V. Error bars correspond to the standard error of the mean.

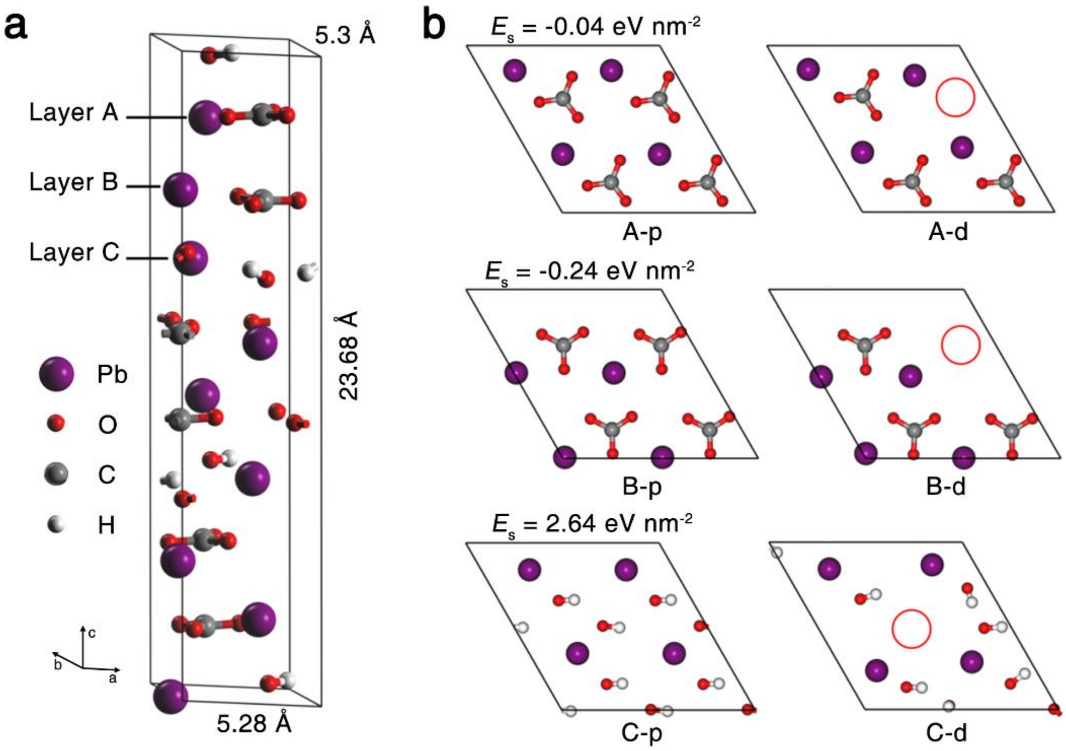

Supplementary Figure 25. Models of hydrocerussite. **a** unit-cell of hydrocerussite. **b (**0001) surfaces with different exposed layers. A-p and A-d donate perfect and defected layer A, respectively, analogously for B and C. E_s represents the surface energy. The purple, red, grey, and white balls are Pb, O, C, and H atoms, respectively. The red circles represent defects.

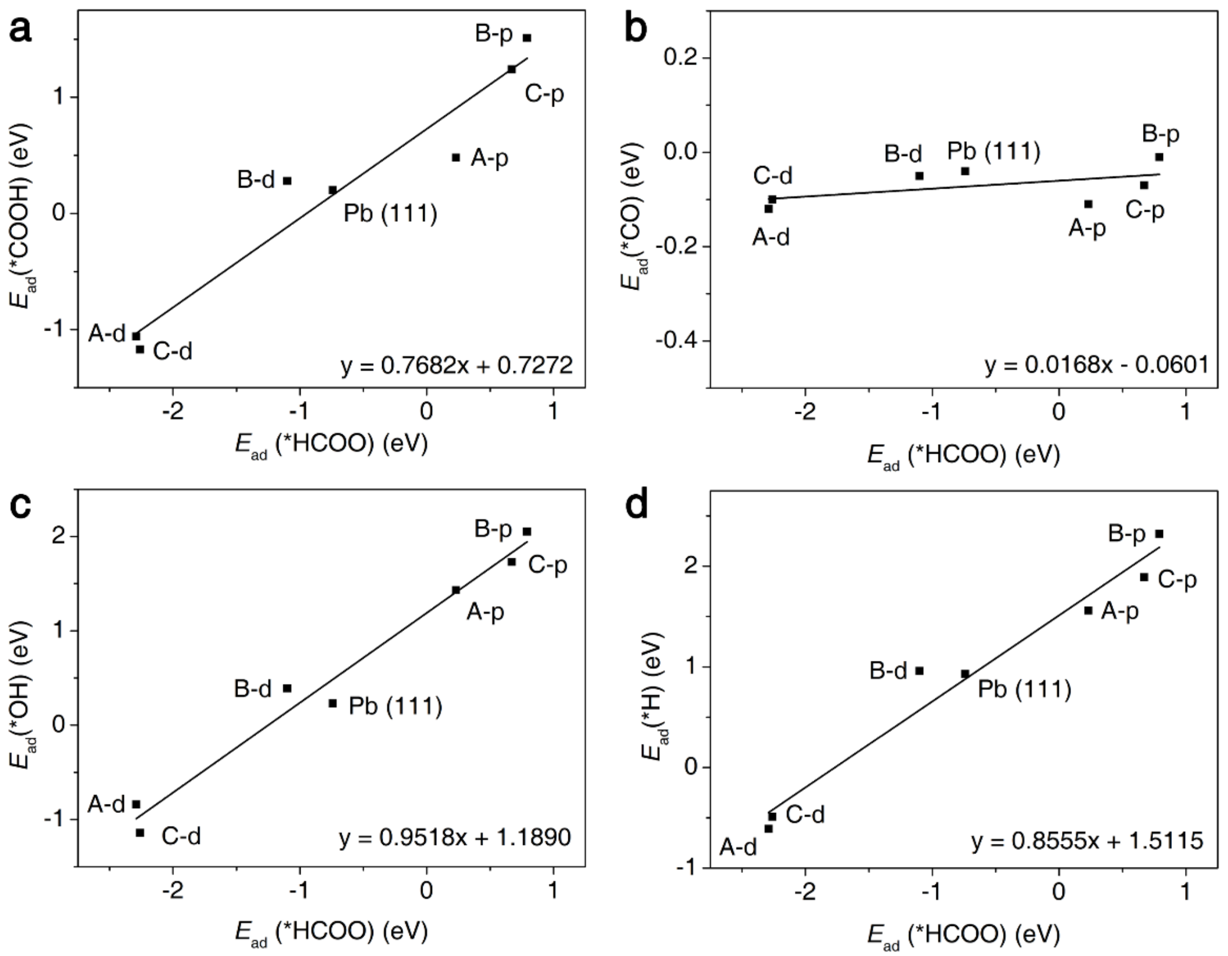

Supplementary Figure 26. Scaling relations between the adsorption energies of involved intermediates and *HCOO over different hydrocerussite surfaces and Pb(111).

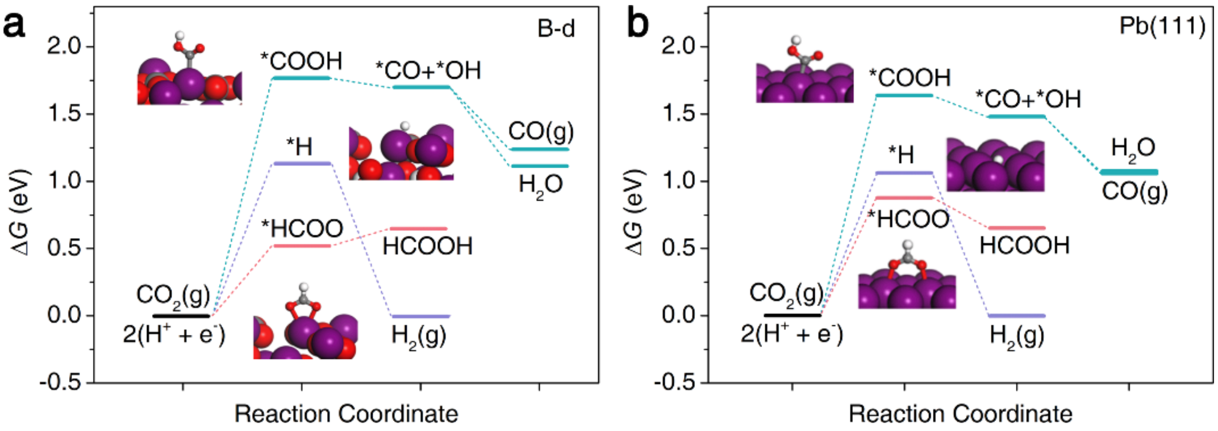

Supplementary Figure 27. Free energy diagrams of HER and CO_2RR at 0 V vs RHE. **a** B-d of hydrocerussite. **b** Pb(111). Inserts are the adsorption configurations of *HCOO, *COOH, and *H. The purple, red, grey, and white balls are Pb, O, C, and H atoms, respectively.

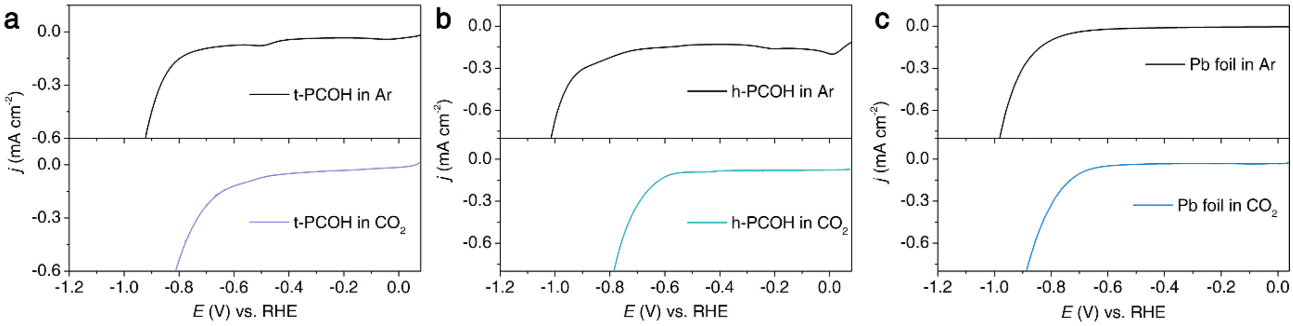

Supplementary Figure 28. Comparison of LSV curves in Ar and CO_2. **a** t-PCOH. **b** h-PCOH. **c** Pb foil.

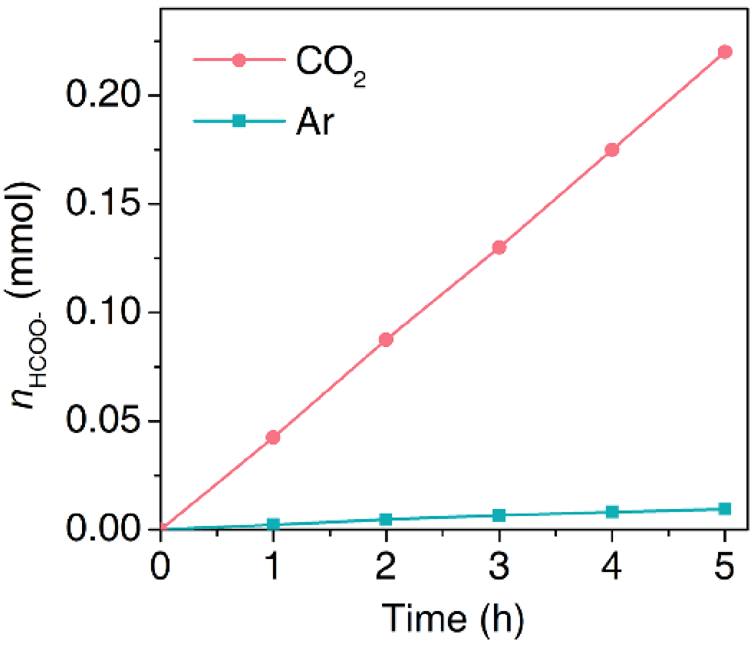

Supplementary Figure 29. Comparison of formate production of h-PCOH at 0.92 V under CO_2 and Ar in 0.5 M $NaHCO_3$. In the absence of CO_2, both the FE and the production of formate shows a sharp decrease. Accordingly, it also can be concluded that the gaseous CO_2 is the primary source for formate generation on hydrocerussite rather than the bicarbonates in solution.

Supplementary Table 1. CO_2RR performance comparison of some formate producing electrocatalysts.

Catalyst	Electrolyte	Optimum potential	*j* (mA cm^{-2})	FE of formate	Ref.
**TA-Pb**	**0.5 M $KHCO_3$**	**-0.92 V vs. RHE**	**-1**	**96.4 %**	**This work**
Roughened Pb	0.1 M $KHCO_3$	-0.96 V vs. RHE	-1.17	88 %	6
$Sn_{56.3}Pb_{43.7}$ alloy	0.1 M K_2SO_4	-2 V vs. Ag/AgCl	-45.7	79.8 %	7
Pb electrode	[Bmim]PF_6-AcN-H_2O	-2.3 V vs. Ag/Ag^+	-37.6	91.6 %	8
PbO_2	[Bzmin]BF_4-H_2O-acetonitrile	-2.3 V vs. Ag/Ag^+	-40.8	95.5 %	9
OD-Pb	0.5 M $NaHCO_3$	-1 V ~ -0.75 V vs. RHE	-0.1~1	~100 %	10
Cubic Pb	0.1 M $KHCO_3$	-1.7 V vs. Ag/AgCl	-	94.1 %	11
Carbon supported Pb	0.5 M $KHCO_3$	-1.94 V vs. RHE	-35	30 %	12
Pb granules	0.5 M $KHCO_3$	-1.5 V vs. SCE	-0.79	90.0 %	13
Pb wire	0.5 M $KHCO_3$	-1.4 V vs. SCE	-2.9	44 %	14
Pb plate	CO_2-saturated 0.5 M NaOH	-1.6 V vs. SCE	-2.5	90 %	15
Etched Pb	0.1 M K_2SO_4	-1.6 V vs. Ag/AgCl	~-0.2	~50 %	16
Pb dendrites	1 M $KHCO_3$	-0.99 V vs. RHE	-7.5	97 %	17
HAS-Bi	0.5 M $KHCO_3$	-1.5 V vs. SCE	-1.4	92 %	18
Bi nanostructure	0.5 M $KHCO_3$	-0.9 V vs. RHE	-15	92 %	19
Bi nanosheets	0.5 M $NaHCO_3$	-1.5 V vs. SCE	-11	95 %	20
SnO_2 nanosheets	0.5 M $NaHCO_3$	-1.6 V vs. Ag/AgCl	-45	87 %	21
SnO_2 porous nanowires	0.1 M $KHCO_3$	-1 V vs. RHE	-10	78 %	22
Sn(S)/Au	0.1 M $KHCO_3$	-0.75 V vs. RHE	-55	93 %	23

Supplementary References

1. Ejima, H., *et al.* One-step assembly of coordination complexes for versatile film and particle engineering. *Science*, **341**, 154-157 (2013).
2. Figueiredo, M. C., Ledezma-Yanez, I. & Koper, M. T. M. In situ spectroscopic study of CO_2 electroreduction at copper electrodes in acetonitrile. *ACS Catal.*, **6**, 2382-2392 (2016).
3. Yu, X., Diao, Q., Zhang, X., Lee, Y.-I. & Liu, H.-G. *In situ* generated Pb nanoclusters on basic lead carbonate ultrathin nanoplates as an effective heterogeneous catalyst. *CrystEngComm*, **19**, 2860-2869 (2017).
4. Gao, D., Zhang, X. & Gao, W. Formation of bundle-shaped beta-$NaYF_4$ upconversion microtubes *via* Ostwald ripening. *ACS Appl. Mater. Inter.*, **5**, 9732-9739 (2013).
5. Li, R., Luo, Z. & Papadimitrakopoulos, F. Redox-assisted asymmetric Ostwald ripening of CdSe dots to rods. *J. Am. Chem. Soc.*, **128**, 6280-6281 (2006).
6. He, Z., *et al.* Electrochemically created roughened lead plate for electrochemical reduction of aqueous CO_2. *Catal. Commun.*, **72**, 38-42 (2015).
7. Choi, S. Y., Jeong, S. K., Kim, H. J., Baek, I.-H. & Park, K. T. Electrochemical reduction of carbon dioxide to formate on tin–lead alloys. *ACS Sustain. Chem. Eng.*, **4**, 1311-1318 (2016).
8. Zhu, Q., *et al.* Efficient reduction of CO_2 into formic acid on a lead or tin electrode using an ionic liquid catholyte mixture. *Angew. Chem. Int. Ed.*, **55**, 9012-9016 (2016).
9. Wu, H., Song, J., Xie, C., Hu, Y. & Han, B. Highly efficient electrochemical reduction of CO_2 into formic acid over lead dioxide in an ionic liquid–catholyte mixture. *Green Chem.*, **20**, 1765-1769 (2018).
10. Lee, C. H. & Kanan, M. W. Controlling H^+ vs CO_2 reduction selectivity on Pb electrodes. *ACS Catal.*, **5**, 465-469 (2014).
11. Kwon, Y. & Lee, J. Formic acid from carbon dioxide on nanolayered electrocatalyst. *Electrocatalysis*, **1**, 108-115 (2010).
12. Kumawat, A. S. & Sarkar, A. Comparative study of carbon supported Pb, Bi and Sn catalysts for electroreduction of carbon dioxide in alkaline medium. *J. Electrochem. Soc.*, **164**, H1112-H1120 (2017).
13. KÖLELI, F., *et al.* Electrochemical reduction of CO_2 at Pb- and Sn-electrodes in a fixed-bed reactor in aqueous K_2CO_3 and $KHCO_3$ media. *J. Appl. Electrochem.*, **33**, 447-450 (2003).
14. Innocent, B., *et al.* FTIR spectroscopy study of the reduction of carbon dioxide on lead electrode in aqueous medium. *Appl. Catal. B-Environ.*, **94**, 219-224 (2010).
15. Innocent, B., *et al.* Electro-reduction of carbon dioxide to formate on lead electrode in aqueous medium. *J. Appl. Electrochem.*, **39**, 227-232 (2008).
16. Pander, J. E., Baruch, M. F. & Bocarsly, A. B. Probing the mechanism of aqueous CO_2 reduction on post-transition-metal electrodes using ATR-IR spectroelectrochemistry. *ACS Catal.*, **6**, 7824-7833 (2016).
17. Fan, M., Garbarino, S., Botton, G. A., Tavares, A. C. & Guay, D. Selective electroreduction of CO_2 to formate on 3D [100] Pb dendrites with nanometer-sized needle-like tips. *J. Mater. Chem. A*, **5**, 20747-20756 (2017).
18. Zhang, H., *et al.* Selective electro-reduction of CO_2 to formate on nanostructured Bi from reduction of BiOCl nanosheets. *Electrochem. Commun.*, **46**, 63-66 (2014).
19. Lu, P., *et al.* Facile synthesis of a bismuth nanostructure with enhanced selectivity for electrochemical conversion of CO_2 to formate. *Nanoscale*, **11**, 7805-7812 (2019).
20. Han, N., *et al.* Ultrathin bismuth nanosheets from *in situ* topotactic transformation for selective electrocatalytic CO_2 reduction to formate. *Nat. Commun.*, **9**, 1320 (2018).
21. Li, F., Chen, L., Knowles, G. P., MacFarlane, D. R. & Zhang, J. Hierarchical mesoporous SnO_2 nanosheets on carbon cloth: A robust and flexible electrocatalyst for CO_2 reduction with high efficiency and selectivity. *Angew. Chem. Int. Ed.*, **56**, 505-509 (2017).

22. Kumar, B., *et al.* Reduced SnO_2 porous nanowires with a high density of grain boundaries as catalysts for efficient electrochemical CO_2-into-HCOOH conversion *Angew. Chem. Int. Ed.*, **56**, 3645-3649 (2017).
23. Zheng, X., *et al.* Sulfur-modulated tin sites enable highly selective electrochemical reduction of CO_2 to formate. *Joule*, **1**, 794-805 (2017).
