# Supplementary material for: Unveiling hydrocerussite as an electrochemically stable active phase for efficient carbon dioxide electroreduction to formate
Source: Nat Commun. 2020 Jul 8;11:3415. doi: 10.1038/s41467-020-17120-9 (PMC7343827; doi:10.1038/s41467-020-17120-9)
Supplement: Supplementary file 2 — Peer Review File [file 41467_2020_17120_MOESM2_ESM.docx]

Peer Review Information

**Journal:** Nature Communications

**Manuscript title:** Self-reduction vs. CO_2_ reduction: unveiling hydrocerussite as an active phase for electrochemical formate generation

**Corresponding author name(s):** Prof Bin Zhang

**Editorial notes:**

**none**

**Reviewer comments & decisions:**

**Reviewer comments, first version:**

Reviewer #1 (Remarks to the Author):

This work addresses the use of a hydrocerussite electrocatalyst (Pb3(CO3)2(OH)2, denoted as t-PCOH) as a selective and stable catalyst for CO2 reduction reaction (CO2 RR). The paper reports faradaic efficiencies (FE) to formate above 96 %. FE remain above 90 % after 10 hours of CO2 electroreduction reduction. This precatalyst (hydrocerussite, t-PCOH hereafter) is obtained sequentially after electrolysis (in the presence of CO2) using a tannin-lead(II) (TA-Pb) complex film deposited on carbon paper.
From a thorough study (based on in situ and ex situ measurements) authors stated that the precatalyst (t-PCOH) is active in the form of nanosheets. They prepared hydrocerussite nanoplates (denoted as h-PCOH) and studied the behaviour of this catalyst in CO2 RR. They found better stability of h-PCOH during electrolysis in the presence of CO2 bubbling than in the presence of Ar bubbling.
They found FE around 90 % with this nanoplate catalyst (h-PCOH), which is higher than that obtained with a Pb foil (~40 %). They attribute the higher FE to the suppression of the self-reduction of the electrocatalyst (Pb II in the form of hydrocerussite) during CO2 RR.
In my opinion the claims reported in the paper are of interest to other researchers in this field (CO2 electroreduction), and probably in the field of electrocatalysts development.
Authors present a wide study with experimental (using advanced techniques) and theoretical calculations. Thus, it can be a guideline for deeper understanding of electrocatalysts behaviour (and its transformation process) during cathodic reactions.
Original results are presented in this paper. However, it would be convenient if authors could include references of works studying Pb electrodes for CO2 electroreduction:

B. Innocent, D. Liaigre, D. Pasquier, F. Ropital, J.-M. Léger, K.B. Kokoh, J. Appl. Electrochem. 39 (2009) 227–232.
Z. He, J. Shen, Z. Ni, J. Tang, S. Song, J. Chen, L. Zhao, Catal. Commun. 72 (2015) 38–42.
J. García, C. Jiménez, F. Martínez, R. Camarillo, J. Rincón, J. Catal. 367 (2018) 72–80.

Authors claim that the high selectivity and stability of the electrocatalyst due to the suppression of the self-reduction of the electrocatalyst (Pb) during CO2 electroreduction.
It would be great if they could show the voltammetry results obtained with a Pb foil compared to those of the precatalyst (t-PCOH) and the catalyst (h-PCOH) in figures S6 and S26, respectively.

The statements made in this work are interesting from the point of view of the search for more selective and stable catalysts. The focus of this paper makes it easier to approach the fundamentals of operation of an electrocatalyst during its use, and that is why I think this work is interesting in the field of catalyst development.

I recommend including some minor revisions which would increase the high quality of the work:
• Regarding Figure S2 in Supplementary Information: Could authors explain the behaviour observed at -0.8 V? It would be expected that H2 FE decreased when passing from -0.9 to -0.8 V, however, it increases from ~11% to 55 %.
• It would be great to see bar errors in Figure S2 (as those shown in Figure S22).
• In page 4 of manuscript turnover frequency (TOF) is reported, could authors include an indication on how do they calculated it?
• In page 6 authors claim that “the cyclic voltammetry (CV) curve of t-PCOH shows no redox peak in the tested range, firmly verifying the stability of hydrocerussite in CO2RR (Figure S6).” However, other works in the literature report voltammetry results with different electrode materials (in CO2 RR) and no redox peak is observed. Therefore, the stability claimed for hydrocerussite is not “exclusive” of this material compared to other.
• In relation to the above, it would be interesting to include a CV curve with Pb foil in the presence of CO2 in Figure S6 (together with that of t-PCOH).
• In page 8, lines 178-179: It is affirmed that “The slightly lower FE of h-PCOH may originate from the poor coverage of the relatively large h-PCOH nanoplates (Figure S18), leaving part of the carbonaceous substrate exposed to give off much H2. The result in turn is a good evidence for the high coverage of in situ formed t-PCOH. Moreover, limited by the high mass loading of h-PCOH, its TOF is calculated to be only 6.8×10-4 s-1, which is 80 times lower than that of t-PCOH, further indicating a high catalytic efficiency of the complex-derived hydrocerussite.” Can authors confirm if both electrodes achieved the same hydrocerussite loading on carbon paper? It would be great if they could give the Pb loading, as in page 4 (line 85 for 3-layer TA-Pb precatalyst).

• Could you compare the TOF or the CO2 conversion rate (mmol/h mg Pb) of t-PCOH and of h-PCOH with those reported in the literature for Pb electrocatalysts?

• It would be interesting to include LSV of Pb foil in the presence of CO2 and Ar in Figure S26.

In page 12 it is said for Depositing TA-Pb complex on substrates: “After sufficient adsorption, 10 mL 9 mg mL-1 tannic acid (TA) solution was quickly added.” Can authors specify the time for “sufficient adsorption”, or the way to test it is achieved?

In page 14, line 335, page 14 it is said that mass loading is 5 mg cm-2 in electrodes. It is referring to t-POCH or also to p-POCH?


Reviewer #2 (Remarks to the Author):

Shi et al present a study on tannin-lead(II) complex as electrocatalysts for the CO electrochemical reaction. After thorough characterization, the authors find that the catalyst is reduced to cerussite (PbCO3), and sequentially to hydrocerussite (Pb3(CO3)2(OH)2). Despite this being an interesting work, I don’t think it has the novelty need to be published in Nature Communications. The authors do not give sufficient insight into the stability of hydocerussite under reaction conditions and fail to compare their results with those of state of the art catalyst. I think there are several points the authors could address to improve their work:

1. The authors claim that oxide derived catalysts are not stable. While is true that such catalysts are reduced under reaction conditions they have also been shown to maintain high faradaic efficiency towards the desired product for reaction times up to 100 hours (see for instance Advanced Energy Materials, 2018, 8(31), Journal of the American Chemical Society, 2012, 134(49), 19969-199721802427, Nature communications 2016, 7, 12123)
2. The authors should compare their results with other catalysts, as it seems to me their results are comparable with those obtained for different formate producing catalyst (see for instance Nanoscale, 2019, 11(16), 7805-7812, Joule, 2017, 1(4), 794-805, Electrochemistry communications, 2014, 46, 63-66).
3. The authors mention the samples where kept under cathodic potential before reaction. Where the catalyst analyzed at this point? As I understand they have reported characterization of the as-prepared sample and after some time of CO2RR. It would be interesting, however, to know the structure of the catalyst after this preconditioning time. It is also not clear for me at what reaction time where PCO and PCOH identified. Could it be that PCO forms during the preconditioning time and PCOH when reacting with CO2?
4. The authors also claim that Pb-TA is not reduced to Pb(0). What was the longest reaction time at which this sample was characterized? How do the authors discard further reduction after 1 hour of reaction?
5. For some catalytic measurements, the reaction time is not given. That is the case for potential and layer optimization (Figure S2). As the authors show a clear time dependence of the catalyst structure this is crucial information. Furthermore, I wonder if the comparison of different potential is fair. Could it be that at certain potentials Pb-TA has not transformed into PCO or PCOH?
6. Where is the carbon to form HCOO- in the CO2 free electrolysis coming from?
7. I find the difference in stability for h-PCOH in the absence of CO2 with respect to that during CO2RR is an interesting observation. In this regard, it would be good to have in the main manuscript some results from h-PCOH to have an easier comparison of the structure of the sample after reaction in Ar and CO2. Furthermore, I think the authors need to discuss the effect of CO2 on the stability of h-PCOH which would give more novel insight into this work.
8. I find the structure of the paper a little odd. Why is the discussion part only focusing on figure 4, which is not previously discussed? Also as I mention I think some results on h-PCOH and c-PCOH should be in the main manuscript to make it easier to follow.


Reviewer #3 (Remarks to the Author):

The current research article is quite interesting due to in-situ conversion of a complex precursor, which serves active phase under CO2 condition. Both experimental and theoretical results were provided to support the study. I suggest following issues which need to be clarified.

Comment 1:
Authors mentioned that, the improvement in FE of TA-PB complex showed highest formate FE of 96.4 at -0.92 V with 3 layers. This is due to extremely low loading of Pb (25 μg cm-2) in that particular layer. Please provide the ICP-MS data for other layered TA-PB complexes in order to further clarify the effect of multiple-layered TA-Pb on catalytic activity.

Comment 2:
Authors provided XRD spectra for the transformation of TA-PB to t-PCHO, but what about elemental compositions. Please clarify about the elemental compositions in TA-PB, t-PCO and t-PCHO (In accordance with XPS results).

Comment 3:
Also, authors reported that the valence state of Pb is +2 in whole electrolysis which proves the stability of hydrocerussite. Did author carried out post analysis of the hydrocerussite to confirm its stability after electrolysis? Please provide the necessary analysis to prove its stability before and after catalytic reaction.

Comment 4:
In the experimental section, 10 mL 10 mM Pb(NO3)2 and 10 mL 9 mg mL-1 tannic acid (TA) solution were used for the synthesis of TA-PB complex. what is the exact concentration of TA to Pb in the as formed TA-PB complex? Meaning, the given concentrations of particular components are optimized or not?

Comment 5:
The authors must add more information about the material and discussion about the advantages/disadvantages of the proposed catalyst.

Comment 6:
The description of electrochemical experiment must be improved. Two points are critical: the necessity in the removal of dissolved oxygen and the description of EIS experiment, specifically equivalent circuit and roughness estimation. Without this information, the following consideration of many details is speculative. The estimation of surface coatings needs the size of the working electrode.

Comment 7:
The influence of specific surface area of as­prepared samples on its electrocatalytic activity should be discussed in detail.

Comment 8:
The electrochemical CO2RR mechanism should be provided in the chemistry aspect. The corresponding discussion or a schematic picture should be provided.

Comment 9:
Please provide the XPS and electrocatalytic result images with clearly.

Comment 10:
What is the effect of scan rate in electrochemical CO2RR ?

Comment 11:
Some of the grammar and style issues: Please check entire manuscript, in many places adjectives are used instead of nouns.

Comment 12:
The preparation of sample is not exhaustive; some parameters are not listed. Such as stirring time and temperature.

Comment 13:
The authors must include the electrochemical work set up with neat diagram which will be interesting to the readers.

**Author rebuttal, first version:**

**To reviewer 1:**

**Reviewer letter:** This work addresses the use of a hydrocerussite electrocatalyst (Pb_3_(CO_3_)_2_(OH)_2_, denoted as t-PCOH) as a selective and stable catalyst for CO_2_ reduction reaction (CO_2_ RR). The paper reports faradaic efficiencies (FE) to formate above 96 %. FE remain above 90 % after 10 hours of CO_2_ electroreduction reduction. This precatalyst (hydrocerussite, t-PCOH hereafter) is obtained sequentially after electrolysis (in the presence of CO_2_) using a tannin-lead(II) (TA-Pb) complex film deposited on carbon paper.

From a thorough study (based on in situ and ex situ measurements) authors stated that the precatalyst (t-PCOH) is active in the form of nanosheets. They prepared hydrocerussite nanoplates (denoted as h-PCOH) and studied the behaviour of this catalyst in CO_2_RR. They found better stability of h-PCOH during electrolysis in the presence of CO_2_ bubbling than in the presence of Ar bubbling.

They found FE around 90 % with this nanoplate catalyst (h-PCOH), which is higher than that obtained with a Pb foil (~40 %). They attribute the higher FE to the suppression of the self-reduction of the electrocatalyst (Pb II in the form of hydrocerussite) during CO_2_ RR.

In my opinion the claims reported in the paper are of interest to other researchers in this field (CO_2_ electroreduction), and probably in the field of electrocatalysts development. Authors present a wide study with experimental (using advanced techniques) and theoretical calculations. Thus, it can be a guideline for deeper understanding of electrocatalysts behaviour (and its transformation process) during cathodic reactions. Original results are presented in this paper. However, it would be convenient if authors could include references of works studying Pb electrodes for CO_2_ electroreduction:

B. Innocent, D. Liaigre, D. Pasquier, F. Ropital, J.-M. Léger, K.B. Kokoh, J. Appl. Electrochem. 39 (2009) 227–232.

Z. He, J. Shen, Z. Ni, J. Tang, S. Song, J. Chen, L. Zhao, Catal. Commun. 72 (2015) 38–42.

J. García, C. Jiménez, F. Martínez, R. Camarillo, J. Rincón, J. Catal. 367 (2018) 72–80.

Authors claim that the high selectivity and stability of the electrocatalyst due to the suppression of the self-reduction of the electrocatalyst (Pb) during CO_2_ electroreduction. It would be great if they could show the voltammetry results obtained with a Pb foil compared to those of the precatalyst (t-PCOH) and the catalyst (h-PCOH) in figures S6 and S26, respectively.

The statements made in this work are interesting from the point of view of the search for more selective and stable catalysts. The focus of this paper makes it easier to approach the fundamentals of operation of an electrocatalyst during its use, and that is why I think this work is interesting in the field of catalyst development.

I recommend including some minor revisions which would increase the high quality of the work:

**Answer:** We sincerely acknowledge the reviewer for the positive evaluation and constructive comments. Accordingly, we have added the three important references to our revised manuscript (cited as Ref. 29-31). Besides, we have added the CV curve and LSV curve of Pb foil as Figure S8 and S28 in our revised ESI. For your convenience, the figures are attached in the following (Figure R1), and the key revisions are highlighted in red font in the revised manuscript and revised ESI.

**Figure R1.** (a) CV curve of Pb foil in CO_2_. (b) LSV curves of Pb foil in Ar and in CO_2_.

**Comment 1.** Regarding Figure S2 in Supplementary Information: Could authors explain the behavior observed at -0.8 V? It would be expected that H_2_ FE decreased when passing from -0.9 to -0.8 V, however, it increases from ~11% to 55 %.

**Answer:** We do appreciate this kind comment. To make it clear, the corresponding partial current density of products based on the results of FE was shown in Figure R2. It can be seen that from -0.82 V to -0.92 V, the partial current density of formate increased from -0.58 to -1.51 mA cm^-2^. However, the partial current density of H_2_ remains nearly constant (about -0.5 mA cm^-2^). The amount of CO production can be neglected. With the partial current density of formate increasing from -0.82 to -0.92 V, while that of H_2_ staying the same, the formate FE increased from 41 to 88 %, and the H_2_ FE decreased from 57 to 11 %.

**Figure R2.** Partial current density of H_2_, formate and CO at different potentials. The columns at -0.82 and -0.92 V are magnified for clear comparison.

**Comment 2.** It would be great to see bar errors in Figure S2 (as those shown in Figure S22).

**Answer:** We do acknowledge this nice suggestion. We have added the error bars in our revised ESI as Figure S3 now. For your convenience, the revised figure is attached as Figure R3 shown as follows.

**Figure R3.** (a) FEs of single-layer TA-Pb at different potentials. (b) FEs of different layers of TA-Pb at the optimum potential of -0.92 V.

**Comment 3.** In page 4 of manuscript turnover frequency (TOF) is reported, could authors include an indication on how do they calculated it?

**Answer:** Thanks for your suggestion. The calculation of TOF is based on equation 1 as follows.

$TOF=\frac{n_{f}}{n_{c}\times t}$	(equation 1)

where *n_f_* is the amount of the generated formate, *n_c_* is the amount of the active catalyst site, and *t* is the duration time. In this work, we assume all the Pb sites are active for the reaction.

The generated formate is calculated according to equation 2.

$n_{f}=\frac{Q\times\mathrm{FE}_{f}}{2\times F}$	(equation 2)

where *Q* is the integrated coulombs from the *I-t* curve of TA-Pb, FE_f_ is the Faradaic efficiency of formate, and *F* is the Faradaic constant.

So based on the above equations, the TOF value of TA-Pb is calculated to be 0.055 s^-1^. We have also added the calculation method of TOF to our revised ESI.

**Comment 4.** In page 6 authors claim that “the cyclic voltammetry (CV) curve of t-PCOH shows no redox peak in the tested range, firmly verifying the stability of hydrocerussite in CO2RR (Figure S6).” However, other works in the literature report voltammetry results with different electrode materials (in CO2 RR) and no redox peak is observed. Therefore, the stability claimed for hydrocerussite is not “exclusive” of this material compared to other.

**Answer:** We acknowledge the kind comment. Generally, a peak appears in the voltammogram means the occurrence of a redox reaction. A well-known example is that the oxidation peak of Ni(II) to Ni(III) can always be found when Ni-based materials are used as the OER electrocatalysts. Similarly, the redox peak of Pb/PbO reversibly has been observed at 0~-0.2 V vs. RHE (***ACS Catal.*** 2016, 6, 7824-7833). In the voltammograms of both t-PCOH and Pb foil, no redox peaks can be observed in this region under CO_2_, indicating that t-PCOH is not reduced to metal Pb under this condition.

**Comment 5.** In relation to the above, it would be interesting to include a CV curve with Pb foil in the presence of CO_2_ in Figure S6 (together with that of t-PCOH).

**Answer:** We acknowledge the kind suggestions of this reviewer. We have added the CV curve of Pb foil in the presence of CO_2_ in our revised ESI, as shown in Figure R1a.

**Comment 6.** In page 8, lines 178-179: It is affirmed that “The slightly lower FE of h-PCOH may originate from the poor coverage of the relatively large h-PCOH nanoplates (Figure S18), leaving part of the carbonaceous substrate exposed to give off much H_2_. The result in turn is a good evidence for the high coverage of in situ formed t-PCOH. Moreover, limited by the high mass loading of h-PCOH, its TOF is calculated to be only 6.8×10^-4^ s^-1^, which is 80 times lower than that of t-PCOH, further indicating a high catalytic efficiency of the complex-derived hydrocerussite.” Can authors confirm if both electrodes achieved the same hydrocerussite loading on carbon paper? It would be great if they could give the Pb loading, as in page 4 (line 85 for 3-layer TA-Pb precatalyst).

**Answer:** Thanks for the constructive suggestion. As shown in the “Electrochemical measurements” in “Methods”, we have shown that the mass loading of h-PCOH is 5 mg cm^-2^. Based on the chemical formula of hydrocerussite (Pb_3_(CO_3_)_2_(OH)_2_), the mass loading of Pb in h-PCOH is calculated to be 4.01 mg cm^-2^. According the reviewer’s suggestion, we have added the mass loading of Pb in h-PCOH to our revised manuscript (page 9, line 13), which is copied as follows.

*“Moreover, limited by the high mass loading of h-PCOH (4.01 mg cm^-2^ for Pb), its TOF is calculated to be only 6.8×10^-4^ s^-1^.”*

**Comment 7.** Could you compare the TOF or the CO_2_ conversion rate (mmol/h mg Pb) of t-PCOH and of h-PCOH with those reported in the literature for Pb electrocatalysts?

**Answer:** We do appreciate the kind suggestion. We have summarized many reports by using Pb-based materials as electrocatalysts for CO_2_ reduction to formate in Table R1. We found that Pb plates or wires with modification were often directly used as the electrode, combined with the very difficult estimation of Pb loading. As a result, the corresponding TOF or CO_2_ conversion rate was rarely reported. Also, some reported conversion rates show different units, so it cannot be directly compared with the conversion rate with the unit of mmol mg^-1^ h^-1^. Only two reports showed the conversion rates with ~0.21 and 0.0038 mmol mg^-1^ h^-1^ for Sn_56.3_Pb_43.7_ alloy and carbon-supported Pb, respectively (red font in Table R1), both of which are lower than our TA-Pb (0.90 mmol mg^-1^ h^-1^). Besides, our material shows almost the highest formate FE among all the Pb-based electrocatalysts. We also added this table to our revised ESI as Table S1.

**Table R1.** CO_2_RR performances comparison with some Pb-based reports.

| Catalyst | Electrolyte | Optimum potential | *j* (mA cm^-2^) | FE | Production rate of formate | Ref. |
| --- | --- | --- | --- | --- | --- | --- |
| TA-Pb | **0.5 M KHCO_3_** | **-0.92 V vs. RHE** | **1** | **96.4 %** | **0.90 mmol mg^-1^ h^-1^** | **This work** |
| Roughened Pb | 0.1 M KHCO_3_ | -0.96 V vs. RHE | 1.17 | 88 % |  | 1 |
| Sn_56.3_Pb_43.7_ alloy | 0.1 M K_2_SO_4_ | -2 V vs. Ag/AgCl | 45.7 | 79.8 % | ~0.21 mmol mg^-1^ h^-1^ | 2 |
| Pb electrode | [Bmim]PF_6_-AcN-H_2_O | -2.3 V vs. Ag/Ag^+^ | 37.6 | 91.6 % |  | 3 |
| PbO_2_ | [Bzmin]BF_4_-H_2_O-acetonitrile | -2.3 V vs. Ag/Ag^+^ | 40.8 | 95.5 % |  | 4 |
| OD-Pb | 0.5 M NaHCO_3_ | -1 V ~ -0.75 V  vs. RHE | 0.1~1 | ~100 % |  | 5 |
| Cubic Pb | 0.1 M KHCO_3_ | -1.7 V vs. Ag/AgCl | - | 94.1 % |  | 6 |
| Carbon supported Pb | 0.5 M KHCO_3_ | -1.94 V vs. RHE | 35 | 30 % | ~0.0038 mmol mg^-1^ h^-1^ | 7 |
| Pb granules | 0.5 M KHCO_3_ | -1.5 V vs. SCE | 0.79 | 90.0 % |  | 8 |
| Pb wire | 0.5 M KHCO_3_ | -1.4 V vs. SCE | 2.9 | 44 % | ~0.84 mmol h^-1^ | 9 |
| Pb plate | CO_2_-saturated  0.5 M NaOH | -1.6 V vs. SCE | 2.5 | 90 % |  | 10 |
| Etched Pb | 0.1 M K_2_SO_4_ | -1.6 V vs. Ag/AgCl | ~0.2 | ~50 % |  | 11 |
| Pb dendrites | 1 M KHCO_3_ | -0.99 V vs. RHE | 7.5 | 97 % | 5.92×10^-7^ M min^-1^ | 12 |

**References**

1.	He, Z.*, et al.* Electrochemically created roughened lead plate for electrochemical reduction of aqueous CO_2_. *Catal. Commun.*, **72**, 38-42 (2015).

2.	Choi, S. Y., Jeong, S. K., Kim, H. J., Baek, I.-H. & Park, K. T. Electrochemical reduction of carbon dioxide to formate on tin–lead alloys. *ACS Sustain. Chem. Eng.*, **4**, 1311-1318 (2016).

3.	Zhu, Q.*, et al.* Efficient reduction of CO_2_ into formic acid on a lead or tin electrode using an ionic liquid catholyte mixture. *Angew. Chem. Int. Ed.*, **55**, 9012-9016 (2016).

4.	Wu, H., Song, J., Xie, C., Hu, Y. & Han, B. Highly efficient electrochemical reduction of CO_2_ into formic acid over lead dioxide in an ionic liquid–catholyte mixture. *Green Chem.*, **20**, 1765-1769 (2018).

5.	Lee, C. H. & Kanan, M. W. Controlling H^+^ vs CO_2_ reduction selectivity on Pb electrodes. *ACS Catal.*, **5**, 465-469 (2014).

6.	Kwon, Y. & Lee, J. Formic acid from carbon dioxide on nanolayered electrocatalyst. *Electrocatalysis*, **1**, 108-115 (2010).

7.	Kumawat, A. S. & Sarkar, A. Comparative study of carbon supported Pb, Bi and Sn catalysts for electroreduction of carbon dioxide in alkaline medium. *J. Electrochem. Soc.*, **164**, H1112-H1120 (2017).

8.	KÖLELI, F.*, et al.* Electrochemical reduction of CO_2_ at Pb- and Sn-electrodes in a fixed-bed reactor in aqueous K_2_CO_3_ and KHCO_3_ media. *J. Appl. Electrochem.*, **33**, 447-450 (2003).

9.	Innocent, B.*, et al.* FTIR spectroscopy study of the reduction of carbon dioxide on lead electrode in aqueous medium. *Appl. Catal. B-Environ.*, **94**, 219-224 (2010).

10.	Innocent, B.*, et al.* Electro-reduction of carbon dioxide to formate on lead electrode in aqueous medium. *J. Appl. Electrochem.*, **39**, 227-232 (2008).

11.	Pander, J. E., Baruch, M. F. & Bocarsly, A. B. Probing the mechanism of aqueous CO_2_ reduction on post-transition-metal electrodes using ATR-IR spectroelectrochemistry. *ACS Catal.*, **6**, 7824-7833 (2016).

12.	Fan, M., Garbarino, S., Botton, G. A., Tavares, A. C. & Guay, D. Selective electroreduction of CO_2_ to formate on 3D [100] Pb dendrites with nanometer-sized needle-like tips. *J. Mater. Chem. A*, **5**, 20747-20756 (2017).

**Comment 8.** It would be interesting to include LSV of Pb foil in the presence of CO_2_ and Ar in Figure S26.

**Answer:** Thanks for the helpful suggestion. We have added the LSV curve of Pb foil in both CO_2_ and Ar in our revised ESI as Figure S28. To save the reviewer’s valuable time, this figure is extracted as Figure R1b as mentioned above.

**Comment 9.** In page 12 it is said for Depositing TA-Pb complex on substrates: “After sufficient adsorption, 10 mL 9 mg mL-1 tannic acid (TA) solution was quickly added.” Can authors specify the time for “sufficient adsorption”, or the way to test it is achieved?

**Answer:** We sincerely acknowledge the nice comment. The typical adsorption time in our case is at least 15 minutes. We have also added the adsorption time in our revised manuscript (Page 13, line 15).

**Comment 10.** In page 14, line 335, page 14 it is said that mass loading is 5 mg cm^-2^ in electrodes. It is referring to t-POCH or also to p-POCH?

**Answer:** We appreciate the careful reading and nice comment. The mass loading of 5 mg cm^-2^ is referring to c-PCO and h-PCOH, not to t-PCOH.

**To reviewer 2:**

**Reviewer letter:** Shi et al present a study on tannin-lead(II) complex as electrocatalysts for the CO electrochemical reaction. After thorough characterization, the authors find that the catalyst is reduced to cerussite (PbCO_3_), and sequentially to hydrocerussite (Pb_3_(CO_3_)_2_(OH)_2_). Despite this being an interesting work, I don’t think it has the novelty need to be published in Nature Communications. The authors do not give sufficient insight into the stability of hydrocerussite under reaction conditions and fail to compare their results with those of state of the art catalyst. I think there are several points the authors could address to improve their work:

**Answer:** We sincerely acknowledge the kind comments. We would like to emphasize the novelty of our work again. In this work, we report a scenario to develop hydrocerussite as a stable and active electrocatalyst *via* *in situ* conversion of a complex precursor, tannin-lead(II) (TA-Pb) complex. By investigating the transformation of TA-Pb under electrochemical CO_2_RR condition, we practically and theoretically unveil hydrocerussite as the highly selective electrocatalyst to convert CO_2_ into formate by dominant CO_2_RR to suppress the reduction of the electrocatalyst itself and accompanying HER.

The novelty and innovation of this work include (1) investigating the transformation of TA-Pb to hydrocerussite under CO_2_RR condition, (2) confirming hydrocerussite as an active and stable electrocatalyst for CO_2_RR; (3) theoretically simulating the CO_2_RR nature on hydrocerussite. This work not only offers a new approach of enhancing the selectivity in CO_2_RR by suppressing the self-reduction of the electrode, but also provides a guideline for studying the reaction mechanism and active phases of electrocatalysts.

It should be pointed out that both the other two reviewers spoke highly of our work. Even this reviewer himself/herself admitted in the review letter and the comments many times that our work was interesting and crucial.

Other reasons for the negative concerns on our work are the stability insight and performance comparison with other reports. Both of that are easy to be addressed and the corresponding answers are provided in the following specific comments.

We hope that the reviewer can find a great improvement in our work’s quality in the new version. To save the reviewer’s valuable time, key revisions are displayed in red font in the revised manuscript and the revised ESI.

**Comment 1.** The authors claim that oxide derived catalysts are not stable. While is true that such catalysts are reduced under reaction conditions they have also been shown to maintain high faradaic efficiency towards the desired product for reaction times up to 100 hours (see for instance Advanced Energy Materials, 2018, 8(31), Journal of the American Chemical Society, 2012, 134(49), 19969-199721802427, Nature communications 2016, 7, 12123)

**Answer:** We do appreciate the kind comment. The original description in our manuscript is that “*It is found the residual metastable metal oxides, such as BiOI, PbO_2_, SnO_2_, CuO, are tightly correlated with the high selectivity in CO_2_RR*”. That indicated that the oxide catalysts are not stable, because they tend to be reduced to zero valence. However, the oxide-derived catalysts, i.e. the mixture of the reduced zero-valence metal and their residual oxides, is stable at the CO_2_RR condition and mainly contribute to the CO_2_RR. The reviewer may misunderstand our statement. So to make it clearer, we revised the sentence to “*Although many high-valence metal electrocatalysts are proved to be reduced to zero-valence metal at CO_2_RR condition, it is found the residual metastable metal oxides, such as BiOI, PbO_2_, SnO_2_, CuO, are tightly correlated with the high selectivity in CO_2_RR.*” in our revised manuscript.

**Comment 2.** The authors should compare their results with other catalysts, as it seems to me their results are comparable with those obtained for different formate producing catalyst (see for instance Nanoscale, 2019, 11(16), 7805-7812, Joule, 2017, 1(4), 794-805, Electrochemistry communications, 2014, 46, 63-66).

**Answer:** We acknowledge the helpful suggestion of this reviewer. We have added the comparison of our results with other formate producing catalysts in our revised ESI as Table S1. For your convenience, the table is attached as follows. The recommended references by the reviewer are also included and shown in red font. From this table, it can be seen that our TA-Pb shows almost the highest formate FE among the current work.

**Table R2.** CO_2_RR performance comparison of some formate producing electrocatalysts.

| Catalyst | Electrolyte | Optimum potential | Formate FE | Ref. |
| --- | --- | --- | --- | --- |
| TA-Pb | **0.5 M KHCO_3_** | **-0.92 V vs. RHE** | **96.4 %** | **This work** |
| Roughened Pb | 0.1 M KHCO_3_ | -0.96 V vs. RHE | 88 % | 1 |
| Sn_56.3_Pb_43.7_ alloy | 0.1 M K_2_SO_4_ | -2 V vs. Ag/AgCl | 79.8 % | 2 |
| Pb electrode | [Bmim]PF_6_-AcN-H_2_O | -2.3 V vs. Ag/Ag^+^ | 91.6 % | 3 |
| PbO_2_ | [Bzmin]BF_4_-H_2_O-acetonitrile | -2.3 V vs. Ag/Ag^+^ | 95.5 % | 4 |
| OD-Pb | 0.5 M NaHCO_3_ | -1 V ~ -0.75 V vs. RHE | ~100 % | 5 |
| Cubic Pb | 0.1 M KHCO_3_ | -1.7 V vs. Ag/AgCl | 94.1 % | 6 |
| Carbon supported Pb | 0.5 M KHCO_3_ | -1.94 V vs. RHE | 30 % | 7 |
| Pb granules | 0.5 M KHCO_3_ | -1.5 V vs. SCE | 90.0 % | 8 |
| Pb wire | 0.5 M KHCO_3_ | -1.4 V vs. SCE | 44 % | 9 |
| Pb plate | CO_2_-saturated  0.5 M NaOH | -1.6 V vs. SCE | 90 % | 10 |
| Etched Pb | 0.1 M K_2_SO_4_ | -1.6 V vs. Ag/AgCl | ~50 % | 11 |
| Pb dendrites | 1 M KHCO_3_ | -0.99 V vs. RHE | 97 % | 12 |
| HAS-Bi | 0.5 M KHCO_3_ | -1.5 V vs. SCE | 92 % | 13 |
| Bi nanostructure | 0.5 M KHCO_3_ | -0.9 V vs. RHE | 92 % | 14 |
| Bi nanosheets | 0.5 M NaHCO_3_ | -1.5 V vs. SCE | 95 % | 15 |
| SnO_2_ nanosheets | 0.5 M NaHCO_3_ | -1.6 V vs. Ag/AgCl | 87 % | 16 |
| SnO_2_ porous nanowires | 0.1 M KHCO_3_ | -1 V vs. RHE | 78 % | 17 |
| Sn(S)/Au | 0.1 M KHCO_3_ | -0.75 V vs. RHE | 93 % | 18 |

**References**

1.	He, Z.*, et al.* Electrochemically created roughened lead plate for electrochemical reduction of aqueous CO_2_. *Catal. Commun.*, **72**, 38-42 (2015).

2.	Choi, S. Y., Jeong, S. K., Kim, H. J., Baek, I.-H. & Park, K. T. Electrochemical reduction of carbon dioxide to formate on tin–lead alloys. *ACS Sustain. Chem. Eng.*, **4**, 1311-1318 (2016).

3.	Zhu, Q.*, et al.* Efficient reduction of CO_2_ into formic acid on a lead or tin electrode using an ionic liquid catholyte mixture. *Angew. Chem. Int. Ed.*, **55**, 9012-9016 (2016).

4.	Wu, H., Song, J., Xie, C., Hu, Y. & Han, B. Highly efficient electrochemical reduction of CO_2_ into formic acid over lead dioxide in an ionic liquid–catholyte mixture. *Green Chem.*, **20**, 1765-1769 (2018).

5.	Lee, C. H. & Kanan, M. W. Controlling H^+^ vs CO_2_ reduction selectivity on Pb electrodes. *ACS Catal.*, **5**, 465-469 (2014).

6.	Kwon, Y. & Lee, J. Formic acid from carbon dioxide on nanolayered electrocatalyst. *Electrocatalysis*, **1**, 108-115 (2010).

7.	Kumawat, A. S. & Sarkar, A. Comparative study of carbon supported Pb, Bi and Sn catalysts for electroreduction of carbon dioxide in alkaline medium. *J. Electrochem. Soc.*, **164**, H1112-H1120 (2017).

8.	KÖLELI, F.*, et al.* Electrochemical reduction of CO_2_ at Pb- and Sn-electrodes in a fixed-bed reactor in aqueous K_2_CO_3_ and KHCO_3_ media. *J. Appl. Electrochem.*, **33**, 447-450 (2003).

9.	Innocent, B.*, et al.* FTIR spectroscopy study of the reduction of carbon dioxide on lead electrode in aqueous medium. *Appl. Catal. B-Environ.*, **94**, 219-224 (2010).

10.	Innocent, B.*, et al.* Electro-reduction of carbon dioxide to formate on lead electrode in aqueous medium. *J. Appl. Electrochem.*, **39**, 227-232 (2008).

11.	Pander, J. E., Baruch, M. F. & Bocarsly, A. B. Probing the mechanism of aqueous CO_2_ reduction on post-transition-metal electrodes using ATR-IR spectroelectrochemistry. *ACS Catal.*, **6**, 7824-7833 (2016).

12.	Fan, M., Garbarino, S., Botton, G. A., Tavares, A. C. & Guay, D. Selective electroreduction of CO_2_ to formate on 3D [100] Pb dendrites with nanometer-sized needle-like tips. *J. Mater. Chem. A*, **5**, 20747-20756 (2017).

13.	Zhang, H.*, et al.* Selective electro-reduction of CO_2_ to formate on nanostructured Bi from reduction of BiOCl nanosheets. *Electrochem. Commun.*, **46**, 63-66 (2014).

14.	Lu, P.*, et al.* Facile synthesis of a bismuth nanostructure with enhanced selectivity for electrochemical conversion of CO_2_ to formate. *Nanoscale*, **11**, 7805-7812 (2019).

15.	Han, N.*, et al.* Ultrathin bismuth nanosheets from *in situ* topotactic transformation for selective electrocatalytic CO_2_ reduction to formate. *Nat. Commun.*, **9**, 1320 (2018).

16.	Li, F., Chen, L., Knowles, G. P., MacFarlane, D. R. & Zhang, J. Hierarchical mesoporous SnO_2_ nanosheets on carbon cloth: A robust and flexible electrocatalyst for CO_2_ reduction with high efficiency and selectivity. *Angew. Chem. Int. Ed.*, **56**, 505-509 (2017).

17.	Kumar, B.*, et al.* Reduced SnO_2_ porous nanowires with a high density of grain boundaries as catalysts for efficient electrochemical CO_2_-into-HCOOH conversion *Angew. Chem. Int. Ed.*, **56**, 3645-3649 (2017).

18.	Zheng, X.*, et al.* Sulfur-modulated tin sites enable highly selective electrochemical reduction of CO_2_ to formate. *Joule*, **1**, 794-805 (2017).

**Comment 3.** The authors mention the samples where kept under cathodic potential before reaction. Where the catalyst analyzed at this point? As I understand they have reported characterization of the as-prepared sample and after some time of CO_2_RR. It would be interesting, however, to know the structure of the catalyst after this preconditioning time. It is also not clear for me at what reaction time where PCO and PCOH identified. Could it be that PCO forms during the preconditioning time and PCOH when reacting with CO_2_?

**Answer:** We do appreciate the kind comments. The preconditioning process is *in situ* conducted in the CO_2_RR cell at -0.92 V vs. RHE under CO_2_ bubbling for 30 min, as described in the section of “Electrochemical measurements”. All the electrochemical performances are recorded after the preconditioning process if without additional description.

*I-t* curve is used to identify the stage of the catalyst transformation. As shown in Figure 1d inset, without preconditioning, the *I-t* curve of a piece of as-prepared TA-Pb can be divided into three different stages, corresponding to the transformation from TA-Pb to t-PCO, t-PCO to t-PCOH, and the CO_2_RR on t-PCOH, respectively. It can be found in this figure that t-PCO forms after electrolysis for 3 min, and the formation of t-PCOH is at about 30 min electrolysis. Guiding by the *I-t* curves, we successfully acquired the corresponding XRD patterns at ~3 min for cerussite and ~30 min for hydrocerussite. *In situ* Raman spectra also confirm the formation of cerussite at about 210 s. We also demonstrate the transformation of as-prepared cerussite nanoparticles to hydrocerussite nanosheets can occur and complete in 30 min, which is in good accordance with the *I-t* curve of TA-Pb.

According to these results, preconditioning for 30 min is enough for the complete transformation in our work.

**Comment 4.** The authors also claim that Pb-TA is not reduced to Pb(0). What was the longest reaction time at which this sample was characterized? How do the authors discard further reduction after 1 hour of reaction?

**Answer:** We acknowledge the reviewer’s kind comments. To confirm the stability of TA-Pb, we lengthen the reaction time to 5 h. The corresponding XRD matches well with hydrocerussite without any impurity, indicating no Pb(0) is formed during the process. And we also added this figure to our revised ESI.

**Figure R4.** XRD pattern of t-PCOH after test at -0.92 V for 5 h. The peaks from substrate are labelled by black stars. This figure is labelled as Figure S5 in the revised ESI.

**Comment 5.** For some catalytic measurements, the reaction time is not given. That is the case for potential and layer optimization (Figure S2). As the authors show a clear time dependence of the catalyst structure this is crucial information. Furthermore, I wonder if the comparison of different potential is fair. Could it be that at certain potentials Pb-TA has not transformed into PCO or PCOH?

**Answer:** We acknowledge the useful comment of this reviewer. As we have mentioned in the “Electrochemical measurements” section, “*Before all CO_2_RR measurements, each sample was tested at -0.92 V for 30 min to achieve the steady state*.” The time of 30 min is based on the study of the *I-t* curve of TA-Pb, as discussed in the answer to comment 3. After 30 min, all the catalyst transformation is complete. So the comparison is fair. For each FE record, the sample is maintained at the specific potential for another 30 min to accumulate enough products. We also added the reaction time to our revised manuscript (page 15, line 11).

**Comment 6.** Where is the carbon to form HCOO^-^ in the CO_2_ free electrolysis coming from?

**Answer:** We acknowledge the kind comment of this reviewer. In the absence of CO_2_, the carbon in formate is from NaHCO_3_ as the electrolyte. Without CO_2_ bubbling, the obtained formate decreased to only 4 %, which means the bubbling CO_2_ is the main carbon source for converting CO_2_ to formate.

**Comment 7.** I find the difference in stability for h-PCOH in the absence of CO_2_ with respect to that during CO_2_RR is an interesting observation. In this regard, it would be good to have in the main manuscript some results from h-PCOH to have an easier comparison of the structure of the sample after reaction in Ar and CO_2_. Furthermore, I think the authors need to discuss the effect of CO_2_ on the stability of h-PCOH which would give more novel insight into this work.

**Answer:** Thanks for the constructive comments. We have added the XRD pattern of h-PCOH after testing in CO_2_ for 5 h in the revised Figure 5b to directly compare with that in Ar. We have also added the discussion on the effect of CO_2_ on the stability of h-PCOH in our revised manuscript (page 12, line 6). For your convenience, the added figure and the discussion are attached as follows.

**Figure R5.** Comparison of XRD patterns of h-PCOH after electrolysis under -0.92 V for 5 h in CO_2_ and in Ar. The peaks from the substrate of CP are labelled by black stars.

*“From the above results, it is sure that the presence of CO_2_ is of great importance to maintain the stability of hydrocerussite under cathodic condition. Without CO_2_, hydrocerussite is gradually decomposed and finally reduced to metal Pb, accompanying with enhanced hydrogen evolution and weakened selectivity of formate production. In other words, the preference of CO_2_RR on hydrocerussite can effectively suppress the self-reduction of the electrocatalyst and the accompanying enhanced HER.”*

**Comment 8.** I find the structure of the paper a little odd. Why is the discussion part only focusing on figure 4, which is not previously discussed? Also as I mention I think some results on h-PCOH and c-PCOH should be in the main manuscript to make it easier to follow.

**Answer:** We do appreciate the useful comment and suggestions. Based on the reviewer’s suggestion, we have removed the heading of “Discussion”, and changed the heading of “Results” to “Results and discussion” in our revised manuscript. We also reorganized our manuscript and added a new figure as Figure 3 to show the characterizations of c-PCO and h-PCOH in our revised manuscript. To save the reviewer’s valuable time, this figure is also shown as follows.

**Figure R6.** (a) TEM image of c-PCO. (b) TEM image of c-PCOH transformed from c-PCO. The inset is the corresponding SAED pattern. Scale bar, 2 1/nm. (c) XRD patterns of c-PCO and c-PCOH. The peaks from substrate are labelled by black stars. (d) SEM image of the as-prepared h-PCOH. (e) XRD patterns of h-PCOH before and after CO_2_RR test for 5 h. The peaks from substrate are labelled by black stars. (f) Comparison of formate FEs of t-PCOH, h-PCOH and Pb foil at different potentials.

**To reviewer 3:**

**Reviewer letter:** The current research article is quite interesting due to in-situ conversion of a complex precursor, which serves active phase under CO_2_ condition. Both experimental and theoretical results were provided to support the study. I suggest following issues which need to be clarified.

**Answer:** We sincerely acknowledge the kind comments from this reviewer. For your convenience, the key revisions are highlighted in red font in the revised manuscript and the revised ESI.

**Comment 1.** Authors mentioned that, the improvement in FE of TA-PB complex showed highest formate FE of 96.4 at -0.92 V with 3 layers. This is due to extremely low loading of Pb (25 μg cm^-2^) in that particular layer. Please provide the ICP-MS data for other layered TA-PB complexes in order to further clarify the effect of multiple-layered TA-Pb on catalytic activity.

**Answer:** We do appreciate the kind suggestion. We have tested the ICP-MS data of TA-Pb with other layers. Based on the results, the Pb loading for different layers is calculated and shown as Figure R7. It can be directly found that Pb loading increases with the layers, thus affecting the formate FE. We also added that to our revised ESI as Figure S3c.

**Figure R7.** Pd loading for TA-Pb with different layers.

**Comment 2.** Authors provided XRD spectra for the transformation of TA-PB to t-PCHO, but what about elemental compositions. Please clarify about the elemental compositions in TA-PB, t-PCO and t-PCHO (In accordance with XPS results).

**Answer:** We acknowledge the kind comment. According to the XPS spectra, the elemental composition of TA-Pb, t-PCO, and t-PCOH on carbon paper (CP) as the substrate is shown as Table R3. The thickness of TA-Pb supported on CP is thick enough (~20 nm, Figure S1). So the elemental composition of TA-Pb is considered to be accurate. However, when TA-Pb transforms into t-PCO and t-PCOH, the thickness will severely decrease. In this way, most of the C and a small amount of O in the composition is from the supported CP, making the elemental composition of t-PCO and t-PCOH from XPS unreliable. But t-PCO and t-PCOH cannot be obtained without the substrate. It is still difficult to get the accurate elemental composition of t-PCO and t-PCOH because of the ultrathin thickness.

**Table R3.** Elemental composition of TA-Pb, t-PCO, and t-PCOH from XPS spectra.

| Sample | C (at %) | O (at %) | Pb (at %) |
| --- | --- | --- | --- |
| TA-Pb | 62.48 | 35.24 | 2.38 |
| t-PCO | 90.23 | 9.25 | 0.52 |
| t-PCOH | 81.66 | 15.82 | 2.52 |

**Comment 3.** Also, authors reported that the valence state of Pb is +2 in whole electrolysis which proves the stability of hydrocerussite. Did author carried out post analysis of the hydrocerussite to confirm its stability after electrolysis? Please provide the necessary analysis to prove its stability before and after catalytic reaction.

**Answer:** We sincerely appreciate the kind suggestion. We tested the SEM images, XRD patterns, XPS spectra, and Raman spectra of h-PCOH after testing at -0.92 V for 5 h. To save the reviewer’s valuable time, the results are shown in Figure R8. All the results indicated the morphology, composition, structure, and valence of Pb unchanged after the electrolysis.

**Figure R8.** Comparison of h-PCOH before and after electrolysis for 5 h. (a-b) SEM images of h-PCOH before (a) and after (b) electrolysis. (c) XPS survey spectra, (d) Pb 4f XPS spectra, (e) XRD patterns, and (f) Raman spectra of h-PCOH before and after electrolysis. The corresponding figures in the revision are Figure S21, S22, 3e, and S23, respectively.

**Comment 4.** In the experimental section, 10 mL 10 mM Pb(NO_3_)_2_ and 10 mL 9 mg mL^-1^ tannic acid (TA) solution were used for the synthesis of TA-PB complex. What is the exact concentration of TA to Pb in the as formed TA-PB complex? Meaning, the given concentrations of particular components are optimized or not?

**Answer:** Thanks for the helpful comment. The structure of tannic acid (TA) is shown in Figure R9. It can be found that TA is a kind of polymer with the esterification of gallic acid as the basic unit. So neither the molecular weight nor the content of oxhydryl in a TA molecular is well-defined. So it is difficult to decide the exact ratio of TA to Pb for the synthesis. In our work, both the addition of Pb(NO_3_)_2_ and TA are excess to ensure the complete coverage of the complex on the substrate.

**Figure R9.** Structure of tannic acid.

**Comment 5.** The authors must add more information about the material and discussion about the advantages/disadvantages of the proposed catalyst.

**Answer:** We appreciate the kind comment and suggestions. Previous reports thought most metal-containing electrocatalysts would be reduced to the zero-valence metal as the real active species for electrochemical CO_2_RR. In our work, we found with the preference of CO_2_RR, the self-reduction of the electrocatalysts and the accompanying HER was suppressed. We developed TA-Pb as a highly efficient CO_2_RR pre-catalyst with high formate selectivity of up to 96.4 %. We estimated the electrochemical performance of TA-Pb including the LSV curves, *I-t* curves, FEs of products at different potentials, stability, and TOF. To verify the carbon source, we also conducted the isotope-labelling test. After that, to investigate the transformation of TA-Pb, we obtained TEM images, HRTEM images, XRD patterns, and XPS spectra of each stage of the transformation of TA-Pb. Electrochemical in situ Raman and ATR-FTIR spectroscopy, and quasi-in-situ XAS spectra were also employed to monitor the transformation. To further study the transformation from TA-Pb to cerussite and hydrocerussite, we had also explicitly synthesized cerussite and hydrocerussite to investigate the transformation under operating conditions and the electrochemical performances. Finally, we practically and theoretically conclude that hydrocerussite is steady under CO_2_RR condition and is the active species of TA-Pb for CO_2_RR. As a result, we believe we have provided enough information about our material in the revised manuscript.

For the advantage of our catalyst, as we have emphasized in our revised manuscript, our TA-Pb pre-catalyst shows a high formate selectivity (96.4±0.9 %) with a high turnover frequency (TOF, 0.055 s^-1^) and excellent stability. The comparison with other reported electrocatalysts towards formate is shown in Table R1 (Table S1 in the revised ESI). The result demonstrates that our catalyst shows almost the highest FE towards formate and the highest CO_2_ conversion rate among these.

The only disadvantage for TA-Pb may be the relatively low working current density because of the ultralow catalyst loading. Readers may think Pb is toxic and not eco-friendly. However, in our work, the Pb-containing solution in the sample preparation was carefully collected and stored in a dedicated container. Once the TA-Pb is prepared, it is stable enough under the operating condition. To verify the stability, we analyzed Pb content in the electrolyte after long-term electrolysis by ICP-MS. The result showed that only 4.418 μg L^-1^ Pb was detected, which was lower than the Chinese Standards for Drinking Water Quality (GB 5749-2006) of 0.01 mg L^-1^ for Pb, indicating that the use of hydrocerussite as electrocatalyst for CO_2_RR was eco-friendly. Both the mentioned description has been shown in the section of “Methods”.

**Comment 6.** The description of electrochemical experiment must be improved. Two points are critical: the necessity in the removal of dissolved oxygen and the description of EIS experiment, specifically equivalent circuit and roughness estimation. Without this information, the following consideration of many details is speculative. The estimation of surface coatings needs the size of the working electrode.

**Answer:** We appreciate the comment and suggestion. Prior to the experiments, CO_2_/Ar was bubbled to our system for more than 30 min to remove the dissolved oxygen and continuously bubbled to the system throughout the test. And we have added the description in our revised manuscript (Page 15, line 3).

However, the EIS experiment didn’t appear in our work. And seldom report on electrochemical CO_2_RR discusses EIS spectra and the equivalent circuit because of the complicated kinetics.

Besides, the roughness estimation is normally obtained through the test of double-layer capacitance (*C*_dl_) to estimate the electrochemical surface area. Then the roughness factor (*R*_f_) is calculated by dividing *C*_dl_ by the ideal planar capacitance of the specific electrode materials. So only the comparison for the same material is meaningful. In our work, only TA-Pb and h-PCOH are used as the CO_2_RR electrocatalysts for comparison with each other. However, the as-prepared h-PCOH nanoplates with the size of several micrometers exhibit very poor dispersity on CP (Figure R8a-b). Most of the *C*_dl_ is assigned to the inactive CP rather than the real catalyst. So it is more reasonable to normalize the electrochemical performance from the catalyst loading rather than the specific area. We calculated the turnover frequency (TOF) to accurately estimate the production of formate per active Pb site.

Finally, carbon paper (CP) is used as the substrate for all the working electrode (no mater for coating or drop-casting) with the exposed area of 1 cm^2^, just as stated in the section of “Electrochemical measurements” in our revised manuscript.

**Comment 7.** The influence of specific surface area of as-prepared samples on its electrocatalytic activity should be discussed in detail.

**Answer:** We do acknowledge the kind comment. We must admit that the specific surface area has a great influence on the electrocatalytic activity. However, as we pointed in the last comment, only TA-Pb and h-PCOH are used as the CO_2_RR electrocatalysts for comparison with each other in our work. The as-prepared h-PCOH nanoplates with the size of several mircometers exhibit very poor dispersity on CP (Figure R8a-b). Most of the calculated specific area is assigned to the inactive CP, rather than the real catalyst. So it is more reasonable to normalize the electrochemical performance from the catalyst loading rather than the specific area. We have calculated the turnover frequency (TOF) to estimate the production of formate per Pb site. The TOF of TA-Pb is calculated to be 0.055 s^-1^, while that of h-PCOH is only 6.8×10^-4^ s^-1^, which is 80 times lower than that of t-PCOH, further indicating a high catalytic efficiency of the complex-derived hydrocerussite.

**Comment 8.** The electrochemical CO_2_RR mechanism should be provided in the chemistry aspect. The corresponding discussion or a schematic picture should be provided.

**Answer:** We do acknowledge the kind suggestion. As shown in Figure R10, there are two paths for the generation of formate. In the result of in situ ATR-FTIR spectra, we captured the adsorption of *HCOO as the intermediate, indicating the formate is generated through the red path in Figure R10 on our catalysts. And this result is in accordance with the previous reports (***ChemSusChem***, 2018, 11, 48-58; ***J. Phys. Chem. C***, 2017, 121, 19767-19773), in which Pb-based materials are also apt to the *HCOO path due to the weak combination between Pb and C. Accordingly, we added this figure to our revised ESI as Figure S13.

**Figure R10.** Reaction paths for formate generation. The dominant path in our work is labelled by red color.

**Comment 9.** Please provide the XPS and electrocatalytic result images with clearly.

**Answer:** We sincerely acknowledge the helpful suggestion. We have improved the quality of our figures in the revised manuscript.

**Comment 10.** What is the effect of scan rate in electrochemical CO_2_RR?

**Answer:** We do appreciate the kind comment. The scan rate is an important parameter in voltammetry. It mainly affects the shape of the redox peaks for all electrochemical reactions, not only in CO_2_RR. The larger the scan rate, the higher and earlier the redox peaks appear. Generally, to get accurate onset overpotential and maximally eliminate the double-layer capacitance, the practical scan rate is always as small as possible. To balance the accuracy and the time spent, the scan rate in our work is 10 mV s^-1^. And we also added this description in our revised manuscript.

**Comment 11.** Some of the grammar and style issues: Please check entire manuscript, in many places adjectives are used instead of nouns.

**Answer:** Thanks for the nice suggestions. We have carefully checked our manuscript. We believe the quality of our manuscript will be greatly improved after the revision.

**Comment 12.** The preparation of sample is not exhaustive; some parameters are not listed. Such as stirring time and temperature.

**Answer:** Thanks for the careful review. After the careful check, we added the missing information to our revised manuscript. The preparation of TA-Pb was at room temperature without stirring for 6 h (Page 13, line 17). And the preparation of c-PCO was also at room temperature (Page 14, line 5).

**Comment 13.** The authors must include the electrochemical work set up with neat diagram which will be interesting to the readers.

**Answer:** We appreciate the wise suggestion. We have added the illustration of our electrochemical set up in our revised Figure S2. To save the reviewer’s valuable time, this figure is extracted as Figure R11 as follows.

**Figure R11.** Illustration of the home-made electrochemical CO_2_RR set up used in this work.

***At the end, we acknowledge again for all the recognization and constructive comments and suggestions from all the reviewers. We are sure that the quality of this work has been greatly improved.***

**Reviewer comments, second version:**

Reviewer #1 (Remarks to the Author):

The authors have addressed the reviewer's comments and indications. The article can be now accepted for publication in Nature Communications.


Reviewer #3 (Remarks to the Author):

Accepted

**Author rebuttal, second version:**

**Reviewer comments, third version:**

**Author rebuttal, third version:**
